# The bacteriology of pleural infection (TORPIDS): an exploratory metagenomics analysis through next generation sequencing

**DOI:** 10.1016/S2666-5247(21)00327-X

**Published:** 2022-04

**Authors:** Nikolaos I Kanellakis, John M Wrightson, Stephen Gerry, Nicholas Ilott, John P Corcoran, Eihab O Bedawi, Rachelle Asciak, Andrey Nezhentsev, Anand Sundaralingam, Rob J Hallifax, Greta M Economides, Lucy R Bland, Elizabeth Daly, Xuan Yao, Nick A Maskell, Robert F Miller, Derrick W Crook, Timothy S C Hinks, Tao Dong, Ioannis Psallidas, Najib M Rahman

**Affiliations:** a Oxford Centre for Respiratory Medicine, Churchill Hospital, Oxford University Hospitals NHS Foundation Trust, Oxford, UK; b Laboratory of Pleural and Lung Cancer Translational Research, Nuffield Department of Medicine, University of Oxford, Oxford, UK; c Chinese Academy of Medical Sciences, China Oxford Institute, Nuffield Department of Medicine, University of Oxford, Oxford, UK; d Respiratory Medicine Unit, Nuffield Department of Medicine, University of Oxford, Oxford, UK; e National Institute for Health Research Oxford Biomedical Research Centre, University of Oxford, Oxford, UK; f Centre for Statistics in Medicine, Nuffield Department of Orthopaedics, Rheumatology and Musculoskeletal Sciences, University of Oxford, Oxford, UK; g Oxford Centre for Microbiome Studies, Kennedy Institute of Rheumatology, University of Oxford, Oxford, UK; h MRC Human Immunology Unit, MRC Weatherall Institute of Molecular Medicine, University of Oxford, Oxford, UK; i Academic Respiratory Unit, University of Bristol Medical School Translational Health Sciences, Bristol, UK; j North Bristol Lung Centre, North Bristol NHS Trust, Bristol, UK; k Institute for Global Health, University College London, London, UK; l Nuffield Department of Medicine, University of Oxford and John Radcliffe Hospital, Oxford, UK; m National Institute of Health Research Oxford Biomedical Research Centre, John Radcliffe Hospital, Oxford, UK

## Abstract

**Background:**

Pleural infection is a common and severe disease with high morbidity and mortality worldwide. The knowledge of pleural infection bacteriology remains incomplete, as pathogen detection methods based on culture have insufficient sensitivity and are biased to selected microbes. We designed a study with the aim to discover and investigate the total microbiome of pleural infection and assess the correlation between bacterial patterns and 1-year survival of patients.

**Methods:**

We assessed 243 pleural fluid samples from the PILOT study, a prospective observational study on pleural infection, with 16S rRNA next generation sequencing. 20 pleural fluid samples from patients with pleural effusion due to a non-infectious cause and ten PCR-grade water samples were used as controls. Downstream analysis was done with the DADA2 pipeline. We applied multivariate Cox regression analyses to investigate the association between bacterial patterns and 1-year survival of patients with pleural infection.

**Findings:**

Pleural infection was predominately polymicrobial (192 [79%] of 243 samples), with diverse bacterial frequencies observed in monomicrobial and polymicrobial disease and in both community-acquired and hospital-acquired infection. Mixed anaerobes and other Gram-negative bacteria predominated in community-acquired polymicrobial infection whereas *Streptococcus pneumoniae* prevailed in monomicrobial cases. The presence of anaerobes (hazard ratio 0·46, 95% CI 0·24–0·86, p=0·015) or bacteria of the *Streptococcus anginosus* group (0·43, 0·19–0·97, p=0·043) was associated with better patient survival, whereas the presence (5·80, 2·37–14·21, p<0·0001) or dominance (3·97, 1·20–13·08, p=0·024) of *Staphylococcus aureus* was linked with lower survival. Moreover, dominance of Enterobacteriaceae was associated with higher risk of death (2·26, 1·03–4·93, p=0·041).

**Interpretation:**

Pleural infection is a predominantly polymicrobial infection, explaining the requirement for broad spectrum antibiotic cover in most individuals. High mortality infection associated with *S aureus* and Enterobacteriaceae favours more aggressive, with a narrower spectrum, antibiotic strategies.

**Funding:**

UK Medical Research Council, National Institute for Health Research Oxford Biomedical Research Centre, Wellcome Trust, Oxfordshire Health Services Research Committee, Chinese Academy of Medical Sciences, and John Fell Fund.

## Introduction

Pleural infection is a severe and complex disease with considerable morbidity and mortality worldwide.^1^ Long hospital admissions and requirement for invasive treatments drive pleural infection health-care costs.^1^, ^2^, ^3^

The mainstay of pleural infection treatment is prompt drainage of the pleural effusion and initiation of antimicrobial therapy.^1^ Antibiotics are usually started empirically with broad-spectrum coverage. Knowledge of the predominant organisms causing pleural infection is pivotal to achieving optimal antimicrobial coverage. However, focused and narrow-spectrum antibiotics are not routinely used in pleural infection because the yield from the current gold standard of pathogen identification (culture-based pathogen detection) is between 40% and 60%, due to previous receipt of antimicrobials or to nutritionally fastidious microorganisms.^4^, ^5^, ^6^

Culture-independent nucleic acid amplification has been developed as a reliable alternative method for pathogen detection. A previous study compared conventional culture of pleural fluid and capillary (Sanger) sequencing of the bacterial 16S rRNA gene.^7^ Next generation sequencing (NGS) of the 16S rRNA gene has been used to characterise the total bacteriome of complex human infections and to elucidate the bacterial interactions within biofilms.^8^, ^9^ To our knowledge, only one pleural infection metagenomics study has used 16S rRNA NGS.^10^

The small number of samples, the use of insensitive tests with inadequate sequencing depth and poor clinical-pathological correlation has to date hampered our capacity to study pleural infection bacteriology. Therefore, knowledge of the landscape of pleural infection microbiology remains incomplete. A better understanding of the total pleural infection microbiome could lead to optimised clinical management and reduce hospital stay, complications from antibiotic use and health-care costs.

**Research in context**

**Evidence before this study**

We searched PubMed on Oct 28, 2021, for published systematic reviews, clinical and preclinical studies, and meta-analysis articles on the topic of pleural infection microbiology with the keywords “pleural infection” AND “microbiology” AND “pleural effusion” AND “16S rRNA”, with no language restrictions. We found 41 published studies that fulfilled these criteria, of which 17 were case reports. The remaining 24 studies had small numbers of samples or used insensitive techniques for pathogen detection. We found only one study where 16S rRNA next generation sequencing was used in a total of 64 samples. Pleural infection is a severe and complex disease with increasing incidence—knowledge of causative bacteriology remains incomplete. The association between bacterial patterns and important clinical outcomes including mortality, duration of hospitalisation, and need for surgery is unclear.

**Added value of this study**

To our knowledge, this is the largest translational metagenomics study of pleural infection in adults to date combining high-throughput discovery with high-quality prospective clinical data. Pleural fluid samples from the largest observational study in pleural infection were subjected to 16S rRNA next generation sequencing to characterise the complete microbial landscape. The identified bacterial patterns were associated with clinically important outcomes. Pleural infection was predominately polymicrobial, with mixed anaerobes and other Gram-negative bacteria dominating community-acquired polymicrobial infection, whereas *Streptococcus pneumoniae* dominated in monomicrobial cases. Infections with anaerobes and bacteria of the *Streptococcus anginosus* group were associated with better survival, whereas *Staphylococcus aureus* and Enterobacteriaceae were linked with higher mortality.

**Implications of all the available evidence**

Knowledge of the underlying biology of pleural infection and bacterial patterns has the potential to improve patients' clinical management and potentially shorten hospital stay, minimise complications from antibiotic use, and reduce health-care costs. Understanding the crosstalk between host and pathogen cells and the interactions between bacteria to form biofilms might contribute to developing non-antibiotic-based treatment options. The establishment and use of a culture-independent method for pathogen identification could lead to informed and faster patient stratification to the most appropriate treatment.

Our study (The Oxford Pleural Infection Metagenomics Studies, TORPIDS) used 16S rRNA NGS analysis of pleural fluid samples from the PILOT study.^11^ Our primary aims were to characterise the identified microbes and their abundance in pleural infection and investigate the association between high-fidelity bacterial patterns and 1-year survival in patients with pleural infection. Moreover, we assessed the association of bacterial patterns with the duration of hospitalisation and need for surgery.

## Methods

### Study design and samples

TORPIDS was a prospective follow-up study of the PILOT trial. 263 pleural fluid specimens were subjected to bacterial DNA extraction (50214, Qiagen, Hilden, Germany) followed by 16S rRNA NGS (MiSeq, Illumina, San Diego, CA, USA). 243 of these samples were from adult patients with confirmed pleural infection and 20 were from patients with a pleural effusion from a non-infectious cause (negative control group, appendix 1 pp 6–7). To estimate the background contamination, we applied the same methods to ten non-template control samples (negative control group, PCR-grade water, 17 000–10 Qiagen, Hilden, Germany; appendix 1 pp 16–17).

For the pleural infection group, we used pleural fluid specimens and clinical data prospectively collected at enrolment for the PILOT clinical trial^11^ (appendix 1 p 6). Pleural fluids were cultured for pathogen detection upon collection at the recruitment centres. The specimens used were from the participating UK centres because of limitations on obtaining clinical samples from other countries. Patients were recruited on identical clinical and laboratory criteria between May 1, 2013, and Jan 1, 2017. Evidence of infection was assessed by the recruiting physician on the basis of fever, elevated peripheral blood white-cell count, or elevated serum inflammatory markers (C-reactive protein). Detailed inclusion and exclusion criteria are described in appendix 1 (p 3).

For the negative control group (20 pleural fluids and ten non-template H_2_O), patients did not have clinical or biochemical evidence of infection or systemic inflammation at the time of pleural aspiration. Negative control pleural fluid samples were selected from the Oxford Radcliffe Pleural Biobank, which is a prospective collection of pleural fluid and blood and pleural biopsy specimens.

Ethical and regulatory approval for the study was obtained from the London—Brighton & Sussex Research Ethics Committee (18/LO/1308). The trial is registered with ClinicalTrials.gov, NCT04569110.

### Analysis of 16S rRNA NGS data

We used the FastQC^12^ pipeline for quality assessment and the DADA2^13^ pipeline for downstream analyses of the raw 16S rRNA NGS data. Amplicon sequence variants were classified taxonomically and were removed if the phylum was missing or if they were identified only in samples of the negative control group. Amplicon sequence variants with the same taxonomy were merged, and those with fewer than 100 reads were removed. The abundance of each bacterium in each sample was calculated as the number of reads of bacterium X in sample Y divided by the total number of reads of sample Y, and then multiplied by 100. Bacteria with less than 1% abundance were removed. Bacteria present in the negative cohort were removed from each of the PILOT samples if their abundance was lower than 10% in the PILOT samples (appendix 1 pp 16–17). Detailed methods are described in appendix 1 (pp 3–4).

### Classification of the identified bacteria

We classified the identified bacteria into nine different groups: anaerobic, Enterobacteriaceae, *Staphylococcus aureus, Streptococcus anginosus* group, *Streptococcus pneumoniae, Mycobacterium*, other Gram-positive bacteria, other Gram-negative bacteria, and not available. Bacteria related to Enterobacteriaceae, *S aureus, S anginosus* group, *S pneumoniae*, and *Mycobacterium* were classified into the corresponding groups. The remaining pathogens that were strictly anaerobic were classified as anaerobic, and the rest were classified either as other Gram positive or other Gram negative. One bacterium with incomplete taxonomy, identified in one sample, was classified as not available.

### Sample classification

To explore the association between outcomes and species abundance, we classified samples into one of five groups on the basis of the abundance of the dominant pathogen (appendix 1 p 8): a monomicrobial group for samples in which the dominant pathogen represented 100% of the sequenced bacterial reads (MM group) and four polymicrobial groups for samples in which the dominant pathogen had an abundance between 1% and fewer than 25% of reads (PM1 group), 25% and fewer than 40% of reads (PM2 group), 40% and fewer than 60% of reads (PM3 group), and 60% and fewer than 100% of reads (PM4 group). These four distinct polymicrobial groups were assigned to explore the association of levels of polymicrobiality with outcome and pathogen.

### Unsupervised hierarchical clustering, correlation, and microbiological distance analyses

To study the association between bacteria and to identify bacterial patterns, we did an unsupervised hierarchical clustering analysis, using the Euclidean distance and the complete-linkage method, with the Pheatmap R package used for graph plotting. Additionally, Uniform Manifold Approximation and Projection (UMAP) analysis was performed.^14^ To investigate the correlation between the different samples and further compare the bacterial patterns, we did a correlation analysis using the Spearman method, with the corrplot R package used for graph plotting. To investigate the β diversity (variation of microbiology between samples), we did a microbiological distance analysis using the weighted UniFrac^15^ method, with the pheatmap R package used for graph plotting.

### Outcomes

The primary outcomes of the study were the characterisation of microbes and their abundance in pleural infections and the association between bacterial patterns and 1-year survival. Secondary outcomes were the associations of bacterial patterns with the duration of hospitalisation and 3-month need for surgery, of dental hygiene with dominance of anaerobes, and of bacterial patterns with community-acquired and hospital-acquired infections. Another secondary outcome was a comparison of molecular versus culture-based techniques for bacterial identification.

### Statistical analysis

We analysed 1-year mortality outcomes using multivariable Cox regression analyses adjusted for the RAPID score^16^ as a continuous variable and graphically presented with Kaplan-Meier plots. The RAPID score predicts survival for patients with pleural infection by use of age, urea and albumin concentrations, hospital-acquired infection, and non-purulence.^11^, ^16^ The need for surgery was treated as a binary variable and analysed with multivariate logistical regression, adjusting for the RAPID score^16^ as a continuous variable. We analysed the length-of-stay outcome using univariate Fine and Gray regression analyses to account for the competing risk of death, and we plotted the cumulative incidence. In the multivariable regression analyses, patients with missing data for the RAPID score were excluded, resulting in the exclusion of 33 patients. We did a logistical regression to assess the association between dental hygiene and dominance in anaerobes. Hazard ratios (HR) with 95% CIs are reported. For the comparison of the bacterial patterns between community-acquired and hospital-acquired infection, we used the Shapiro-Wilk and Mann–Whitney–Wilcoxon tests to assess normality and significance. P values lower or equal to 0·05 were considered significant. A specific power analysis was not done for the purposes of this metagenomic study. However, it was previously done for the PILOT^11^ clinical study to show robustness of the RAPID criteria, which that study did. Analyses were done with R (version 3).

### Role of the funding source

The funder of the study had no role in study design, data collection, data analysis, data interpretation, or writing of the report.

## Results

In total, we identified 245 different bacterial species from 243 PILOT study samples (table 1). Anaerobic bacteria exhibited the highest mean abundance (33·5% of all bacterial reads, SD 38·2) followed by other Gram-negative bacteria (27·5%, 34·0) and bacteria of the *S anginosus* group (11·0%, 25·8). Species of the genera *Fusobacterium, Prevotella, Porphyromonas*, and *Parvimonas*, which have all been reported in the oral cavity and dental microbiome,^10^, ^17^ were the most abundant anaerobic bacteria. Anaerobic bacteria were detected in 165 (68%) of 243 PILOT samples and other Gram-negative bacteria were detected in 143 (59%) samples, whereas 70 (29%) samples were positive for *S anginosus* group bacteria. Other Gram-positive bacteria were identified in 73 (30%) samples, albeit with low abundance (6·9%, SD 19·9). Enterobacteriaceae were present in 52 (21%) samples and showed a mean abundance of 8·0% (23·9), with *Escherichia coli* and *Klebsiella* spp being the most common. *S pneumoniae* was detected in 31 (13%) samples, with a mean abundance of 10·5% (29·4).

**Table 1:** Overall pleural infection bacterial microbiology as identified by 16S rRNA next generation sequencing

	**Number of pathogens**	**Mean abundance**	**Number of samples**
Anaerobic	55	33·5% (38·2)	165
Enterobacteriaceae	14	8·0% (23·9)	52
*Staphylococcus aureus*	1	2·6% (13·4)	12
*Streptococcus anginosus* group*	3	11·0% (25·8)	70
*Streptococcus pneumoniae*	1	10·5% (29·4)	31
Other Gram-positive	59	6·9% (19·9)	73
Other Gram-negative	110	27·5% (34·0)	143
*Mycobacterium tuberculosis*	1	0 (0·3)	3
Not available	1	0 (0·1)	1

The detected pathogens were classified into nine groups. The table presents the number of pathogens, mean relative abundance, and number of samples for each bacterial group.

* Consisting of *S anginosus, Streptococcus intermedius*, and *Streptococcus constellatus*.

**Figure 1:**
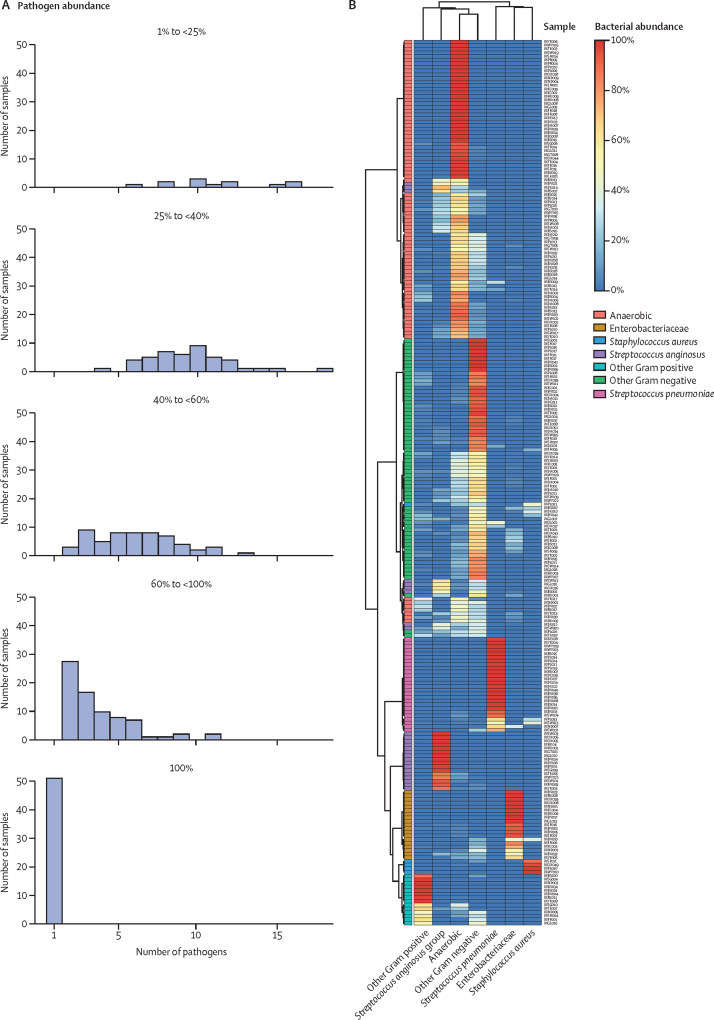
Pleural infection is predominately a polymicrobial disease (A) The histograms show the counts of detected pathogens in each sample within each of the five groups, based on dominant pathogen abundance. (B) Heatmap of unsupervised hierarchical clustering using the Bray-Curtis distance and the complete-linkage method; the colour of the first column denotes the bacterial class of the most abundant pathogen in the sample; each row is a sample and each column is a pathogen group; the colour of each cell represents the abundance (proportion of bacterial reads) of each pathogen group in each sample; red represents high and dark blue low abundance.

Most pleural infection samples were polymicrobial (figure 1A): 16S rRNA NGS detected two or more pathogens in 192 (79%, PM1 to PM4 groups) of 243 samples, and a single pathogen in the other 51 samples (21%, MM group). We detected diverse patterns of polymicrobial pleural infection; polymicrobial groups showed greater species richness (α diversity) compared with that of the MM group (appendix 1 p 8). Unsupervised hierarchical clustering, correlation analyses, and UMAP detected diverse clusters of samples (figure 1B, appendix 1 pp 18–19). The projection showed a closer clustering of samples dominated by *S pneumoniae*. Additionally, we observed two clusters of samples dominated by anaerobic bacteria, the first distant and the second close to samples dominated by other Gram-negative bacteria (appendix 1 p 18). Moreover, distance analysis (β diversity) showed different patterns of microbiological communities between the samples (appendix 1 p 20).

**Figure 2:**
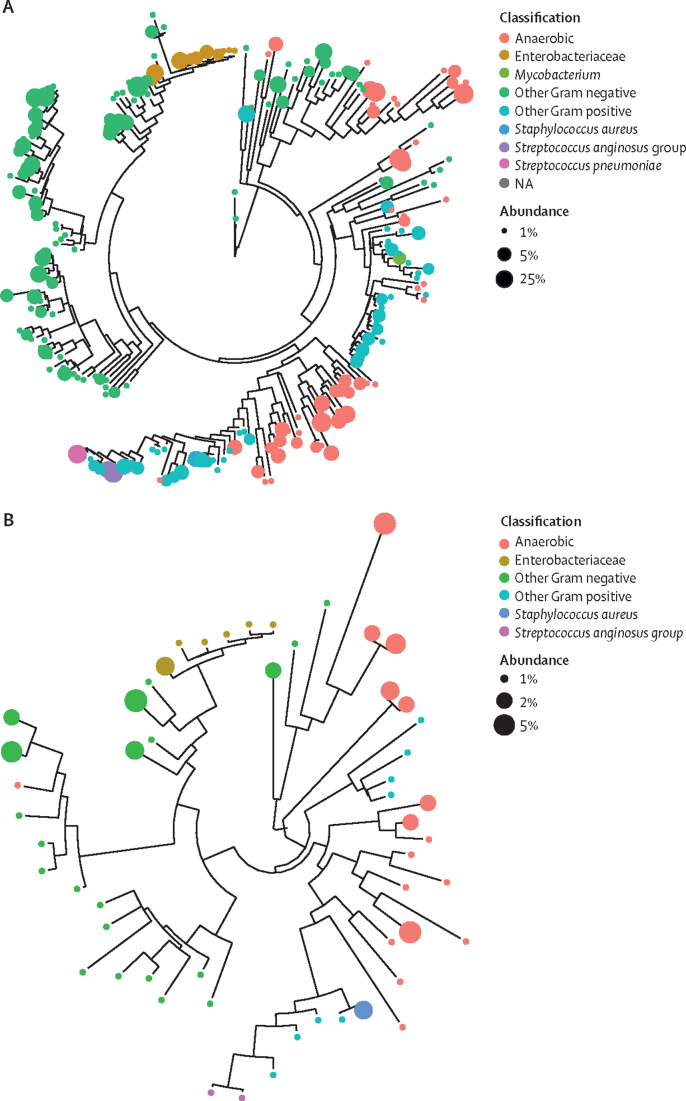
Community-acquired and hospital-acquired pleural infections show distinct bacterial patterns Phylogeny trees of community-acquired (A) and hospital-acquired (B) pleural infections. The colours of the circles represent the pathogen class and the size represents their relative abundance. NA=not available.

Of 243 pleural fluid samples from the PILOT study, 221 (91%) were from patients with a community-acquired infection, 17 (7%) were from those with hospital-acquired infection and five (2%) had an infection of unknown source. We detected diverse bacterial patterns in community-acquired and hospital-acquired pleural infection, as previously described.^7^, ^18^ Compared with hospital-acquired pleural infection, community-acquired infection showed a higher abundance of *S pneumoniae* (p=0·049)*.* Patients with hospital-acquired pleural infection showed a three-times (p=0·043) higher abundance of Enterobacteriaceae and five-times (p=0·0060) higher abundance of *S aureus* (appendix 1 pp 21–22, appendix 2). The abundance of anaerobic (p=0·30) and other Gram-negative (p=0·48) bacteria was similar in patients with community-acquired and hospital-acquired pleural infection. We identified 233 different pathogens in samples from community-acquired pleural infection, showing greater species richness than that of hospital-acquired infection, with 55 different pathogens (figure 2, appendix 1 pp 9–10; appendix 2). We detected no significant differences in the number of identified pathogens per sample between community-acquired (median 4·0, IQR 2·0–8·0) and hospital-acquired (5·0, 2·0–8·0) pleural infection (p=0·65).

**Figure 3:**
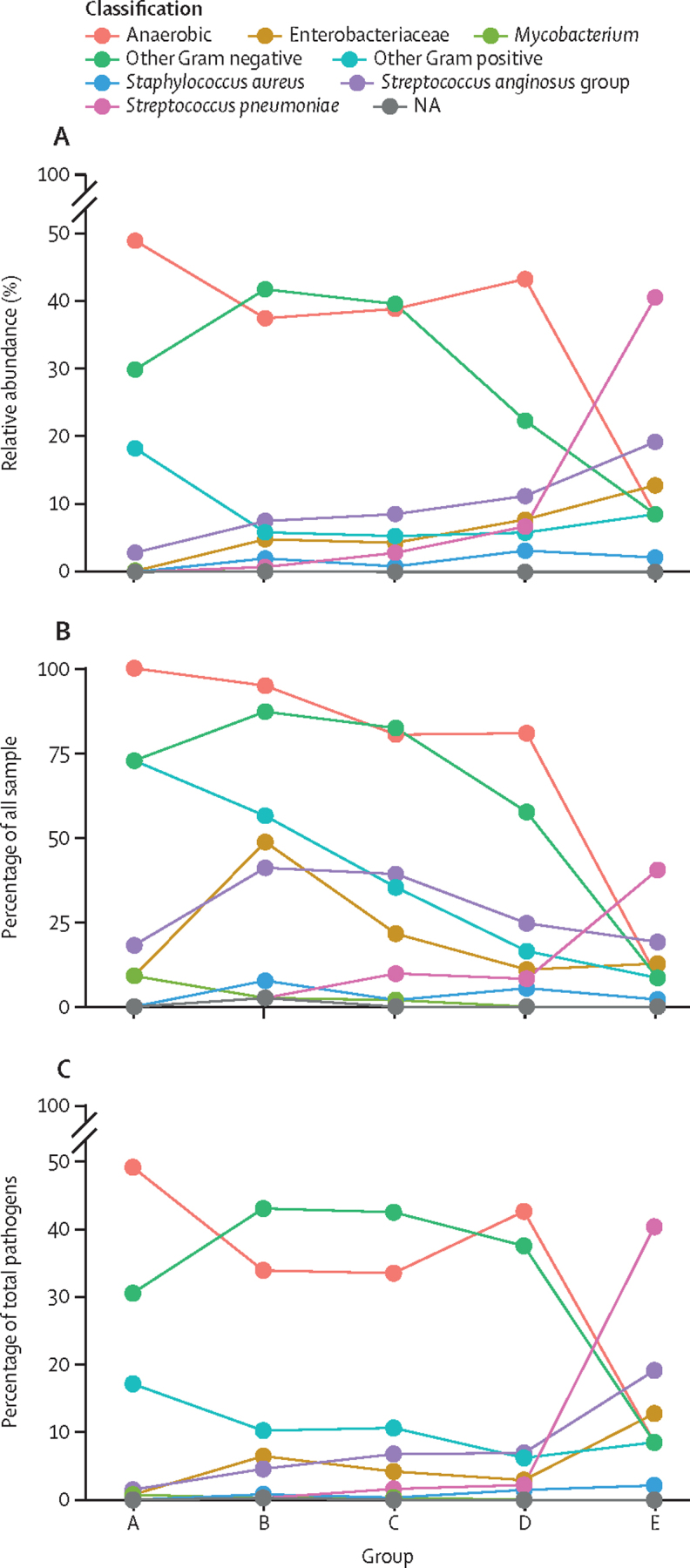
Monomicrobial and polymicrobial community-acquired pleural infections exhibit diverse bacterial patterns Individual dots are joined by a line exclusively to aid readability. (A) Line plot showing the average abundance of each pathogen class per group for community-acquired pleural infections. (B) Line plot showing the frequency (%) of samples containing pathogens of each bacterial class per sample group. (C) Line plot presenting the frequency (%) of each bacterial class per sample group. NA=not available.

We identified distinct and different bacterial patterns in patients with polymicrobial (174 [79%]) and monomicrobial (47 [21%]) community-acquired pleural infection (figure 3). Anaerobic (mean 40·9%, SD 37·7) and other Gram-negative (32·1%, 33·3) bacteria were the most abundant pathogens in PM groups (figure 3A). Of the 174 samples from PM groups, 16S rRNA NGS detected anaerobic bacteria in 148 (85%) samples and other Gram-negative bacteria in 126 (72%) samples (figure 3B). Overall, of the total 1089 bacteria detected in the PM groups, 413 (38%) were anaerobic and 436 (40%) were other Gram-negative bacteria (figure 3C). Additionally, of the bacteria detected in the PM groups, 111 (10%) were other Gram-positive bacteria, which were identified in 60 (27%) of the PM group samples; however, their abundance was low (mean 6·4%, SD 16·8). PM groups showed a species richness of 230 different pathogens.

*S pneumoniae* was the most abundant pathogen (mean 40·4%, SD 49·6) in the MM group, by contrast with all PM groups, which showed a low abundance of *S pneumoniae* (3·8%, 16). In the MM group, the mean abundance was 19·1% (39·8) for the *S anginosus* group and 12·8% (33·7) for Enterobacteriaceae. The MM group showed the lowest species richness, with 12 different bacterial pathogens identified by 16S rRNA NGS (appendix 1 pp 9, 23–24).

Conventional, culture-based pathogen detection was successful in 55 (22%) of 243 samples (appendix 1 p 6), which is lower than that reported in clinical practice and might be due to previous use of antibiotics.^1^ No pathogen was identified by culture in 188 (78%) of 243 samples. Of those samples in which a pathogen was positively identified by culture, one pathogen was detected in 49 (89%) of 55 samples and two pathogens in six (11%). The yield was lower (mean 1·1 pathogens, SD 0·3) compared with 16S rRNA NGS detection, where the mean was 3·2 (2·8) pathogens per sample.

Conventional culture identified bacteria of the *S anginosus* group as the most frequently identified pathogen (15 [27%] of 55 samples), followed by Enterobacteriaceae (12 [22%] samples) and anaerobes (nine [16%] samples). The molecular technique detected anaerobes in 22 (40%) samples, other Gram-negative bacteria in 20 (36%) samples, and *S anginosus* group in 18 (33%) samples (appendix 1 pp 11, 25–27).

A comparison of conventional culture with 16S rRNA NGS showed that 16S rRNA NGS identified the pathogen detected by culture in 48 (87%) of 55 samples. In seven (13%) samples, 16S rRNA NGS did not corroborate culture findings (appendix 1 p 25). However, for five of these seven samples, 16S rRNA NGS detected a bacterium with the same taxonomy (up to the genus level) as the one identified by culture; qPCR assays confirmed the 16S rRNA NGS results (appendix 1 p 28).

We sought to investigate the association between the presence of a bacterial group and patient 1-year survival (table 2, appendix 1 pp 29–30). We did a multivariate Cox regression analysis adjusting for the RAPID score factors, and bacteria were found to be independent predictors of mortality. The presence of anaerobes (HR 0·46, 95% CI 0·24–0·86, p=0·015) and bacteria of the *S anginosus* group (0·43, 0·19–0·97, p=0·043) was associated with better 1-year survival compared with their absence. The presence of *S aureus* was associated with significantly poorer survival (5·80, 2·37–14·21, p<0·0001). No survival differences were detected for other bacterial groups. We examined samples grouped on the basis of the dominant pathogen and 1-year survival using a multivariate Cox regression analysis adjusting for the RAPID score factors. Dominance of *S aureus* (3·97, 1·20–13·08, p=0·024) and Enterobacteriaceae (2·26, 1·03–4·93, p=0·041) were independently associated with a poorer survival than that of samples in which they were not dominant. As previously shown,^7^ hospital-acquired pleural infection had higher mortality than community-acquired pleural infection (3·50, 1·54–7·93, p=0·003; table 2, appendix 1 p 31).

**Table 2:** Cox regression analysis of 1-year risk of mortality

		**Hazard ratio (95% CI)**	**p value**
Presence *vs* absence of			
	Anaerobes	0·46 (0·24–0·86)	0·015
	Enterobacteriaceae	1·50 (0·56–2·36)	0·70
	Other Gram-negative	0·64 (0·34–1·19)	0·16
	Other Gram-positive	0·99 (0·51–1·93)	0·97
	*Staphylococcus aureus*	5·80 (2·37–14·21)	<0·0001
	*Streptococcus anginosus* group*	0·43 (0·19–0·97)	0·043
	*Streptococcus pneumoniae*	1·00 (0·39–2·56)	1·00
Dominance of			
	Anaerobes	0·64 (0·29–1·42)	0·27
	Enterobacteriaceae	2·26 (1·03–4·93)	0·041
	Other Gram-negative	1·06 (0·54–2·11)	0·86
	Other Gram-positive	1·39 (0·43–4·51)	0·58
	*Staphylococcus aureus*	3·97 (1·20–13·08)	0·024
	*Streptococcus anginosus* group*	0·15 (0·02–1·10)	0·062
	*Streptococcus pneumoniae*	1·11 (0·39–3·12)	0·84
Hospital-acquired *vs* community-acquired pleural infection		3·50 (1·54–7·93)	0·0038

Hazard ratios of 1-year death (multivariate Cox regression analysis adjusted for the factors of the RAPID score) comparing the presence versus the absence of each bacterial group, the dominance of each bacterial group against the rest, and hospital-acquired versus community acquired pleural infection.

* Consisting of *S anginosus, Streptococcus intermedius*, and *Streptococcus constellatus*.

We detected no significant relationship between the presence of a bacterial group and the requirement for surgery within 3 months of diagnosis or duration of hospitalisation (appendix 1 pp 12–13). No association was detected between a crude measure of dental hygiene (clinically reported dental status) and predominance of anaerobes (appendix 1 p 14).

## Discussion

In this study, we used 16S rRNA NGS to discover and rigorously investigate the total microbiome of pleural infection and correlate bacterial patterns with prospectively collected and documented clinical outcomes. Our findings suggest that pleural infection is predominately polymicrobial, distinct microbial patterns exist in both monobacterial and polybacterial disease, and the type of bacterial cause is an independent predictor of 1-year survival outcomes.

The incidence of polymicrobial pleural infection has previously been estimated at approximately 23%;^19^ however, this is likely to be an underestimate. Previous studies relied on conventional culture-dependent pathogen detection methods, which have high false-negative rates. Several NGS-based metagenomics studies have indicated that human polymicrobial infections are common.^20^, ^21^

Previous reports based on cultures suggest that aerobic Gram-positive bacteria are the dominant pathogens in community-acquired pleural infection.^19^ Our data contrast with this and showed that anaerobes and Gram-negative bacteria, detected by 16S rRNA NGS, had the highest abundance. This difference might be explained by the fact that anaerobic bacteria are harder to culture with conventional methods and might indicate that the hypoxic environment of the pleural space benefits anaerobic growth.

Increasing evidence exists that bacterial biofilm matrices act as scaffolds that facilitate the attachment of specific bacteria while impeding others.^22^ *S pneumoniae* was the most prevalent pathogen in community-acquired monomicrobial infections—indicating that *S pneumoniae* biofilms might not favour symbiosis with other bacterial species due to strong competition, or that they might not require symbiosis due to sufficient intrinsic virulence factors. By contrast, community-acquired polymicrobial groups were characterised by a prevalent mixture of other Gram-negative and anaerobic bacteria, suggesting possible complex bacterial crosstalk within biofilms and an active process of bacterial co-aggregation in the pathogenesis and evolution of pleural infection.

The mixed pattern of bacterial species within individual pleural infection bacterial niches might point to their cause. The most abundant pleural anaerobic pathogens identified in our study, and in a previous study, are commonly found in the oral cavity and dental microbiome.^10^ Members of the *S anginosus* group are part of the normal oral flora, and *S pneumoniae* is known to colonise the upper respiratory tract. Combined, these data suggest that aspiration of oropharyngeal and oral and dental pathogens might play a substantial role in the aetio-pathogenesis of pleural infection.

The presence of anaerobic and *S anginosus* group bacteria were associated with significantly better survival, whereas survival with *S aureus* infection was poorer, and these results were independent of the only known predictive score for pleural infection survival (RAPID).^11^, ^16^ Patients with a dominance of *S aureus* and Enterobacteriaceae in samples were at higher risk of death, perhaps due to these bacteria being more resistant to antibiotics. This suggests a need for more focused treatment in these patients.

We observed a greater species richness (α diversity) in samples from patients with community-acquired pleural infection compared with those with hospital-acquired disease. This could be explained by exposure of pathogens in the health-care setting to a wider range of antibiotics or disinfectants, resulting in a stronger selection pressure than that faced in community settings.

A comparison of culture and 16S rRNA NGS showed that 16S rRNA NGS had a better yield and shorter turnaround time for results. Whereas pathogen detection in pleural fluid by culture is limited to one or two species, NGS has the potential to identify the complete microbiome.^23^ However, quality assurance protocols are required. US Food and Drug Administration guidelines provide regulatory guidance for NGS-based pathogen diagnostics.^24^ Workflows specifically designed for microbiome analysis are available,^13^, ^25^ yet it remains a challenge to clinically interpret metagenomic data.^9^ Standardised methods for integration of NGS metagenomic information into clinical practice are not widely available^9^, ^26^ but are being rapidly developed.^27^ Emerging long-read sequencing technologies hold the promise of allowing accurate diagnosis at the species level.^28^ The cost of routine 16S rRNA NGS testing might be prohibiting for some centres, but PCR-based pathogen detection panels with lower cost are readily available and clinically validated.^29^

The strengths of our study include its objective design, a large number of well characterised samples, and its link to relevant prospectively collected and highly complete clinical data.^11^ 16S rRNA NGS is an unbiased discovery strategy with the ability to resolve sample polymicrobiality.

Our study also has several limitations. 16S rRNA NGS does not always have optimal resolution up to the species level (ie, it cannot fully differentiate the bacteria of the *S anginosus* group) and cannot differentiate the pathogens that are driving the disease from bystanding bacteria. Available pleural fluid specimens were all from the UK recruitment centres participating in the PILOT study. Geographical location has been associated with infectious pathogens in pleural infection.^19^ The cohort of the PILOT trial were adults, and thus a separate study is required to investigate paediatric pleural infection. The role of viruses was not explored in this study. Finally, due to the processing procedure of clinical samples, we cannot comment on detection of intracellular pathogens although, epidemiologically, these have previously only rarely been described in pleural infection.^7^

In conclusion, our metagenomic assessment of pleural infection samples showed frequent polymicrobiality and clear association of 1-year survival with the type of bacterial cause. Future studies should focus on the relationship between early diagnostics with metagenomic techniques and the effect on clinical outcomes and the association between radiology and the microbiome.

## Data sharing

NGS data are available at the Sequence Read Archive of the US National Center for Biotechnology Information, BioProject ID PRJNA665994.

## Declaration of interests

IP works for AstraZeneca in a non-related field. TSCH reports grants from the Wellcome Trust and The Guardians of the Beit Fellowship, during the conduct of the study; personal fees from AstraZeneca, TEVA, and Peer Voice; grants from Pfizer, National Institute for Health Research (NIHR) Oxford Biomedical Research Centre, University of Oxford, Sensyne Health, and Kymab, outside the submitted work. RFM reports personal fees from Gilead, outside the submitted work. All other authors declare no competing interests.
