# Supplementary material for: The bacteriology of pleural infection (TORPIDS): an exploratory metagenomics analysis through next generation sequencing
Source: Lancet Microbe. 2022 Apr;3(4):e294–302. doi: 10.1016/S2666-5247(21)00327-X (PMC8967721; doi:10.1016/S2666-5247(21)00327-X)
Supplement: Supplementary appendix 1 [file mmc1.pdf]

THE LANCET
Microbe

Supplementary appendix

This appendix formed part of the original submission and has been peer reviewed. We post it as supplied by the authors.

Supplement to: Kanellakis NI, Wrightson JM, Gerry S, et al. The bacteriology of pleural infection (TORPIDS): an exploratory metagenomics analysis through next generation sequencing. *Lancet Microbe* 2022; published online March 11. https://doi.org/10.1016/S2666-5247(21)00327-X.

Supplementary Information

The bacteriology of pleural infection using next generation sequencing: The Oxford Pleural Infection Metagenomics Studies (TORPIDS)

Nikolaos I. Kanellakis[1,2,3,4,*], John M. Wrightson[1,*], Stephen Gerry[5], Nicholas Ilott[6], John P. Corcoran[1], Eihab O. Bedawi[1], Rachelle Asciak[1,2], Andrey Nezhentsev[2], Anand Sundaralingam[1], Rob J. Hallifax[1], Greta M. Economides[2], Lucy R. Bland[2], Elizabeth Daly[2], Xuan Yao[4,7], Nick A. Maskell[8,9], Robert F. Miller[10], Derrick W. Crook[11,12], Timothy S. C. Hinks[1,3,13], Tao Dong[4,7], Ioannis Psallidas[1,2], and Najib M. Rahman[1,2,3,4]

Affiliations:

1. Oxford Centre for Respiratory Medicine, Churchill Hospital, Oxford University Hospitals NHS Foundation Trust, Oxford, OX3 7LE, United Kingdom
2. Laboratory of Pleural and Lung Cancer Translational Research, Nuffield Department of Medicine, University of Oxford, Oxford, OX3 7FZ, United Kingdom
3. National Institute for Health Research Oxford Biomedical Research Centre, University of Oxford, Oxford, OX3 7LE, United Kingdom
4. Chinese Academy of Medical Sciences China Oxford Institute, Nuffield Department of Medicine, University of Oxford, OX3 7FZ, United Kingdom
5. Centre for Statistics in Medicine, Nuffield Department of Orthopaedics, Rheumatology and Musculoskeletal Sciences, University of Oxford, Oxford, OX3 7LD, United Kingdom
6. Oxford Centre for Microbiome Studies, Kennedy Institute of Rheumatology, University of Oxford, Oxford, OX3 7FY, United Kingdom
7. MRC Human Immunology Unit, MRC Weatherall Institute of Molecular Medicine, University of Oxford, Oxford, OX3 9DS United Kingdom
8. Academic Respiratory Unit, University of Bristol Medical School Translational Health Sciences, Bristol, BS10 5NB, United Kingdom
9. North Bristol Lung Centre, North Bristol NHS Trust, Bristol, BS10 5NB, United Kingdom
10. Institute for Global Health, University College London, London, WC1N 6JB, United Kingdom
11. Nuffield Department of Medicine, University of Oxford, John Radcliffe Hospital, Oxford, OX3 9DU, United Kingdom
12. National Institute of Health Research Oxford Biomedical Research Centre, John Radcliffe Hospital, Oxford, OX3 9DU, United Kingdom
13. Respiratory Medicine Unit, Nuffield Department of Medicine, University of Oxford, Oxford, OX3 9DU, United Kingdom

* Equal authors

Corresponding author:

Nikolaos I. Kanellakis PhD

Laboratory of Pleural and Lung Cancer Translational Research, Nuffield Department of Medicine, University of Oxford, Oxford, OX3 7FZ, UK

Email: nikolaos.kanellakis@ndm.ox.ac.uk

Table of Contents

Supplementary Methods 3
Supplementary Tables 6
Supplementary Figures... 16
References.. 34

Supplementary Methods

Inclusion and exclusion criteria for the "Prospective validation of the RAPID clinical risk prediction score in adult patients with pleural infection: the PILOT study" clinical trial

Patients were included if they had a clinical presentation consistent with pleural infection as stated in the Methods section of the main manuscript and any of the following criteria:

1. Pleural fluid that was macroscopically purulent, OR
2. Pleural fluid that was positive on culture for bacterial infection, OR
3. Pleural fluid that demonstrated bacteria on Gram staining, OR
4. Pleural fluid with pH ≤7.2 (measured in a blood gas analyser) or low glucose level (≤3mmol/L
1. or ≤55mg/dL) in a patient with clinical evidence of infection, OR
5. Contrast-enhanced CT evidence of pleural infection (consolidation of underlying lung with enhancing pleural collection) in a patient with clinical evidence of infection, alongside exclusion of other sources of infection.

Exclusion criteria for the PILOT study:

1. Age less than 18 years, OR
2. No pleural fluid available for analysis, OR
3. Patient previous pneumonectomy on the side of pleural infection, OR
4. Expected survival less than three months due to co-morbid disease, as judged by the recruiting physician.

Sample and data collection for the "Prospective validation of the RAPID clinical risk prediction score in adult patients with pleural infection: the PILOT study" clinical trial

Pleural fluid and blood specimens were collected at enrolment. The samples were centrifuged at 400g for 15 minutes at room temperature. Supernatants were transferred into sterile 2.0 ml cryovial microcentrifuge tubes and stored in -80 ^{o}C freezers.

Data collected for each study participant included age, smoking status, dental hygiene, comorbidities, chest radiographic findings, dates of hospital admission and discharge, treatment, culture microbiology findings, haemoglobin, C-reactive protein (CRP), full blood count, pleural fluid LDH, and pleural fluid protein. Survival data for each patient was collected from the trial database and cross-validated with the UK national registry. Patients with missing data were excluded from the analysis.

Bacterial DNA extraction

Bacterial DNA from pleural fluid samples was extracted using the QIAamp UCP Pathogen Mini Kit 50214 (50214, Qiagen, Hilden, Germany) as described elsewhere[1]. DNA quantity and quality were measured by NanoDrop 2000/2000c (ND-2000C, ThermoFisher Scientific, MA, US).

16S rRNA amplicon library preparation and next generation sequencing

Library preparation and sequencing was performed at the Oxford Genomics Centre (OGC). DNA samples were normalised to 12.4ng in 2.5μl volume. Primers targeting the V3-V4 region of the 16S rRNA gene were used for amplification[2]. The full-length primer sequences, using standard International Union of Pure and Applied Chemistry (IUPAC) nucleotide nomenclature, were: 16S Amplicon PCR Forward Primer: 5'-TCGTCGGCAGCGTCAGATGTGTATAAGAGACAGCCTACGGGNGGCWGCAG-3' 16S Amplicon PCR Reverse Primer: 5'-GTCTCGTGGGCTCGGAGATGTGTATAAGAGACAGGACTACHVGGGTATCTAATCC-3'. Following a post-amplicon PCR clean up, we applied a second indexing PCR using NexteraXT indices. Following the second PCR clean-up, libraries were then pooled and diluted into a 10nM final multiplex library ready for sequencing on Illumina MiSeq (San Diego, CA, US).

Analysis of 16S rRNA NGS data

Analysis of raw NGS data, and taxonomy assignment. The quality of raw sequencing data was assessed using FastQC[3] (v0.11.07). Following quality assessment, downstream processing was performed using DADA2,[4] which was implemented in pipeline_dada2.py from the NGSKit repository (https://github.com/nickilott/NGSKit). The filterAndTrim function in DADA2 was used to truncate the forward and reverse reads to 290bp and 225bp, respectively. Retained primer sequences were removed from forward (17bp) and reverse (21bp) reads which resulted in final read lengths of 273bp and 204bp. Error learning, de-replication and sample inference were performed using DADA2 with default parameters. Taxonomy was assigned to amplicon sequence variants (ASVs) using the assignTaxonomy function in DADA2 and the NCBI RefSeq training data (https://zenodo.org/record/2541239/files/RefSeq-RDP16S_v2_May2018.fa.gz).

Analysis of identified ASVs. ASVs were removed if a) they were not assigned to a phylum (n=1,145) or b) they were found only in the thirty samples (Supplementary Table 2) of the negative cohort (n=865). Subsequently, ASVs with the same taxonomy were merged, leading to 561 different species. Post merging, ASVs with fewer than 100 reads (n=198) were removed. The abundance (over bacterial reads) was calculated (see below) for each ASV in each sample and ASVs with less than 1% abundance (n=82) were removed. There were samples which had ASVs with the same taxonomy up to genus level, but not identifiable species. For these ASVs without specification, the number of reads was summed and assigned to the ASV with the highest number of reads in the same genus. ASVs of the negative cohort were removed from each of the PILOT samples if their abundance was less than 10% in the PILOT samples.

Bacterial abundance calculation

To calculate the abundance of bacterium X in sample Y we used the formula below: [reads of bacterium X in sample Y / total reads of sample Y] * 100. The sum of the bacterial abundances in a sample is 100.

In a similar manner to calculate the abundance of bacterial class X in group Y we used the formula below:

[total reads of bacterial class X in group Y/ total reads of group Y] * 100. The sum of the bacterial class abundances in each group is 100.

qPCR assays to cross examine 16S rRNA NGS and culture microbiology results

The qPCR assays were performed using TaqMan® Fast Advanced Master Mix (4444557, ThermoFisher Scientific, MA, US) in a LightCycler® 480 Instrument II (05015243001, Roche, CH). Triplicate samples were used. The qPCR reaction mix, and run method were as per ThermoFisher Scientific protocol. The primers are listed in Supplementary Table 11.

A specific power analysis was not performed for the purposes of this metagenomic study but was performed for the PILOT clinical study to demonstrate robustness of the RAPID criteria, which that study did.

Supplementary Tables

Supplementary Table 1
Demographic characteristics of the pleural infection study participants (PILOT)

Demographic Characteristics		Missing data
Age (years) median, IQR	61 [47, 71]	15 (6%)
Sex	Male 178 (73%)<br>Female 61 (25%)	4 (2%)
Pleural infection aetiology*	Community 221 (91%)<br>Hospital 17 (7%)	5 (2%)
	Monomicrobial 51 (21%)<br>Polymicrobial 192 (79%)	
Hospitalisation duration (days) median, IQR	14 [8, 20]	8 (4%)
Positive culture-based microbiology	Yes 55 (22%)<br>No 181 (74%)	9 (4%)
Surgical drainage	Yes 63 (26%)<br>No 171 (70%)	9 (4%)
Comorbidities	Yes 153 (63%)<br>No 85 (35%)	4 (2%)
Smoking Status	Current smoker 64 (27%)<br>Ex-smoker 107 (44%)<br>Never smoked 65 (27%)	5 (2%)
Dental Hygiene**	Poor 113 (47%)<br>Good 117 (48%)	13 (5%)

* = Hospital acquired disease defined as any hospital contact within a week of diagnosis of pleural infection
** = visually assessed by the recruiting investigator, addressing dental caries and gingivitis

Supplementary Table 2

Demographic characteristics of pleural infection negative study participants

Demographic Characteristics	
Age (years) median, IQR	71 [54, 86]
Sex	Male 11 (73%)<br>Female 9 (25%)
Pleural effusion aetiology	Malignant pleural effusion 10<br>Heart failure 7<br>Hepatic failure 3
White cell count (10^9 cells/L) median, IQR	5·3 [3·1, 6·2]
C-reactive protein (mg/L) median, IQR	11·6 [5·4, 17·9]

Supplementary Table 3
Sample categories

Group	Dominant pathogen abundance (dpa) range	n (%)	Species richness
Polymicrobial 1 - PM 1	1% ≤ dpa < 25%	13 (5·3%)	86
Polymicrobial 2 - PM 2	25% ≤ dpa < 40%	45 (18·5%)	146
Polymicrobial 3 - PM 3	40% ≤ dpa < 60%	58 (23·9%)	111
Polymicrobial 4 – PM 4	60% ≤ dpa < 100%	76 (31.3%)	79
Monomicrobial - MM	100%	51 (21%)	13

The samples were categorized into five classes based on the dominant pathogen abundance (dpa) of the sequenced bacterial reads. The table presents the range of dpa, the number of samples and the species richness for each group. Polymicrobial groups showed greater species richness compared with the monomicrobial group.

Supplementary Table 4
The number of samples and species' richness for each group of community acquired pleural infection.

Group	n (%)	Species richness
Polymicrobial 1 - PM 1	11 (5%)	83
Polymicrobial 2 - PM 2	39 (17·6%)	135
Polymicrobial 3 - PM 3	51 (23·1%)	96
Polymicrobial 4 – PM 4	73 (33%)	79
Monomicrobial - MM	47 (21·3%)	12

Supplementary Table 5
The number of samples and species' richness for each group of hospital acquired pleural infection.

Group	n (%)	Species richness
Polymicrobial 1 - PM 1	2 (11·8%)	12
Polymicrobial 2 - PM 2	2 (11·8%)	18
Polymicrobial 3 - PM 3	7 (41·2%)	36
Polymicrobial 4 – PM 4	3 (17·6%)	7
Monomicrobial - MM	3 (17·6%)	3

Supplementary Table 6
Comparison between culture and 16S rRNA NGS microbiology findings. The table presents the number of pathogens per bacterial group as identified by culture and 16S rRNA NGS for the 55 culture positive samples.

**Bacterial group**	**Culture**	**16S rRNA NGS**
Anaerobic	9	22
Enterobacteriaceae	12	13
Staphylococcus aureus	5	3
Streptococcus anginosus Group	15	18
Streptococcus pneumoniae	6	9
Other Gram-positive	8	8
Other Gram-negative	5	20

Supplementary Table 7
Multivariable logistic regression analyses of the requirement for surgery within three months post diagnosis.

Comparison	Variable	Hazard Ration 95% CI	P value
Presence vs absence of	Anaerobic	0·93 CI [0·44, 1·97]	0·86
	Enterobacteriaceae	0·95 CI [0·41, 2·25]	0·91
	Other Gram-negative	1·49 CI [0·75, 2·98]	0·26
	Other Gram-positive	0·92 CI [0·42, 2·01]	0·84
	Staphylococcus aureus	8,306,256·6 CI [0, Inf]	0·99
	Streptococcus anginosus group	0·87 CI [0·41, 1·85]	0·73
	Streptococcus pneumoniae	0·83 CI [0·31, 2·19]	0·70
Dominance of	Anaerobic	0·99 CI [0·48, 2·05]	0·99
	Enterobacteriaceae	0·42 CI [0·14, 1·28]	0·13
	Other Gram-negative	2·19 CI [0·87, 5·51]	0·10
	Other Gram-positive	0·98 CI [0·21, 4·66]	0·98
	Staphylococcus aureus	2550952·96 CI [0, Inf]	0·99
	Streptococcus anginosus group	0·54 CI [0·20, 1·46]	0·22
	Streptococcus pneumoniae	0·88 CI [0·30, 2·52]	0·81
Acquisition (hospital, community)		1·17 CI [0·25, 5·57]	0·84

The table presents the multivariable logistic regression analyses with adjust for the RAPID score of requirement for surgery within three months post diagnosis comparing a) the presence versus the absence of each bacterial group, b) the dominance of each bacterial group and c) hospital versus community acquired pleural infection. No significant differences were detected between the groups. In the multivariable logistic regression analyses patients with missing data for the RAPID score were excluded from the analyses, resulting in the exclusion of 33 patients.

Supplementary Table 8
Univariate Fine and Gray regression analyses accounting for the competing risk of death did not detect and association between bacterial patterns and the duration of hospital stay.

Comparison	Variable	Sub-distribution hazard ratio (95% CI)	P value
Presence vs absence of	Anaerobic	1·08 CI [0·92, 0·80]	0·60
	Enterobacteriaceae	1·00 CI [0·80, 1·28]	0·98
	Other Gram-negative	1·20 CI [0·91, 1·60]	0·20
	Other Gram-positive	0·96 CI [0·75, 1·24]	0·78
	Staphylococcus aureus	0·72 CI [0·51, 1·01]	0·06
	Streptococcus anginosus group	1·05 CI [0·81, 1·37]	0·69
	Streptococcus pneumoniae	0·94 CI [0·71, 1·26]	0·69
Dominance of	Anaerobic	0·92 CI [0·71, 1·19]	0·52
	Enterobacteriaceae	1·10 CI [0·76, 1·60]	0·60
	Other Gram-negative	1·07 CI [0·79, 1·45]	0·66
	Other Gram-positive	0·97 CI [0·50, 1·89]	0·93
	Staphylococcus aureus	0·80 CI [0·52, 1·23]	0·30
	Streptococcus anginosus group	1·14 CI [0·78, 1·68]	0·51
	Streptococcus pneumoniae	0·93 CI [0·63, 1·39]	0·73
Acquisition (hospital vs community)		1·00 CI [0·72, 1·37]	0·98

The table presents the univariate Fine and Gray regression analyses with adjust for the RAPID score of the duration of hospital stay comparing a) the presence versus the absence of each bacterial group, b) the dominance of each bacterial group against dominance in anaerobes and c) hospital versus community acquired pleural infection. No significant differences were detected between the groups. In the univariate Fine and Gray regression analyses patients with missing data for the RAPID score were excluded from the analyses, resulting in the exclusion of 33 patients.

Supplementary Table 9
Logistic regression analysis of the association between poor dental hygiene and dominance in anaerobes. No significant association was detected.

**Variable**	**Odds Ratio 95% CI**	**P value**
Poor dental hygiene	0·80 CI [0·47, 1·38]	0·43

Supplementary Table 10
qPCR primers

Bacterium target	Gene Target	Name	Sequence	Product length
Streptococcus pneumoniae	lytA	Step-lytA-For	5’-CGTTGACCCTTATCCATATCTTGCT-3	73 bp
		Step-lytA-Rev	5’-GCCGTTCTCAATATCATGCTTAAACTG-3’	
		Step-lytA-Probe	5’-CTCACGGCTAATGCC-3’	
Serratia marcescens	luxS	Ser-luxS-For	5’-TGTGGCCAAATATGCATCTCTGAT-3’	86 bp
		Ser-luxS-Rev	5’-CTTTTCCAACCCTGCGAGTC-3’	
		Ser-luxS-Probe	5’-AAGACGCGCATTTGTT-3’	
Micrococcus luteus	rpf	Micro-Rpf-For	GCCGACGCCACCGA	62 bp
		Micro-Rpf-Rev	GCCTGGCGCTGCAC	
		Micro-Rpf-Probe	TCGAGCGCACCTCCTC	
Universal	16S rRNA	Provided by Thermoscientific, Cat ID Hs03003631_g1		

Supplementary Figures

Supplementary Figure 1

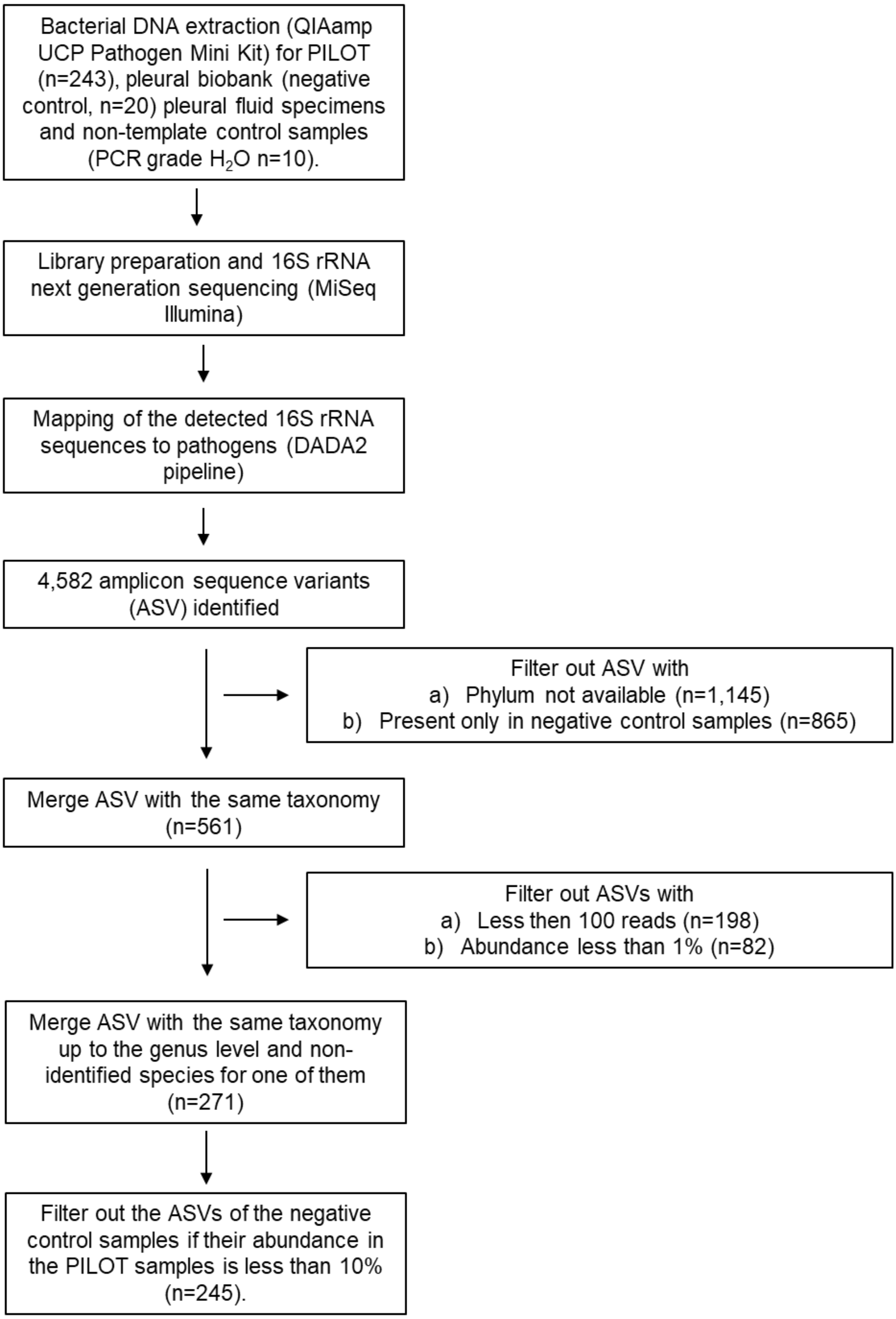

Supplementary Figure 1. Flow diagram of pleural fluid specimen sequencing and analysis.

Pleural fluid samples from the PILOT study (n=243) and non-pleural infection patients obtained from Oxford Pleural Biobank (negative control, n=20) and ten non-template PCR grade H_2O were subjected to bacterial DNA extraction (QIAamp UCP Pathogen Mini Kit, Qiagen) and 16S rRNA sequencing (MiSeq, Illumina). DADA2 pipeline identified 4,582 amplicon sequences variants (ASVs). ASVs with not available phylum (n=1,145) or present only in the negative control samples (n=865) were excluded. Different ASVs with the same taxonomy were merged resulting to 561 unique ASVs. For each sample, pathogens with less than 100 reads (n=198) or less than 1% relative abundance were removed (n=82) by reassigning their reads to zero. For each sample ASVs with the same taxonomy up to the genus level and with no available species for one of them were merged, leading to 271 unique ASVs. The reads of the ASVs of the negative control samples were filtered out from each of the PILOT samples if their abundance was less than 10% (in the PILOT samples). Finally, 245 ASVs were subjected to further analyses.

Supplementary Figure 2

A

B

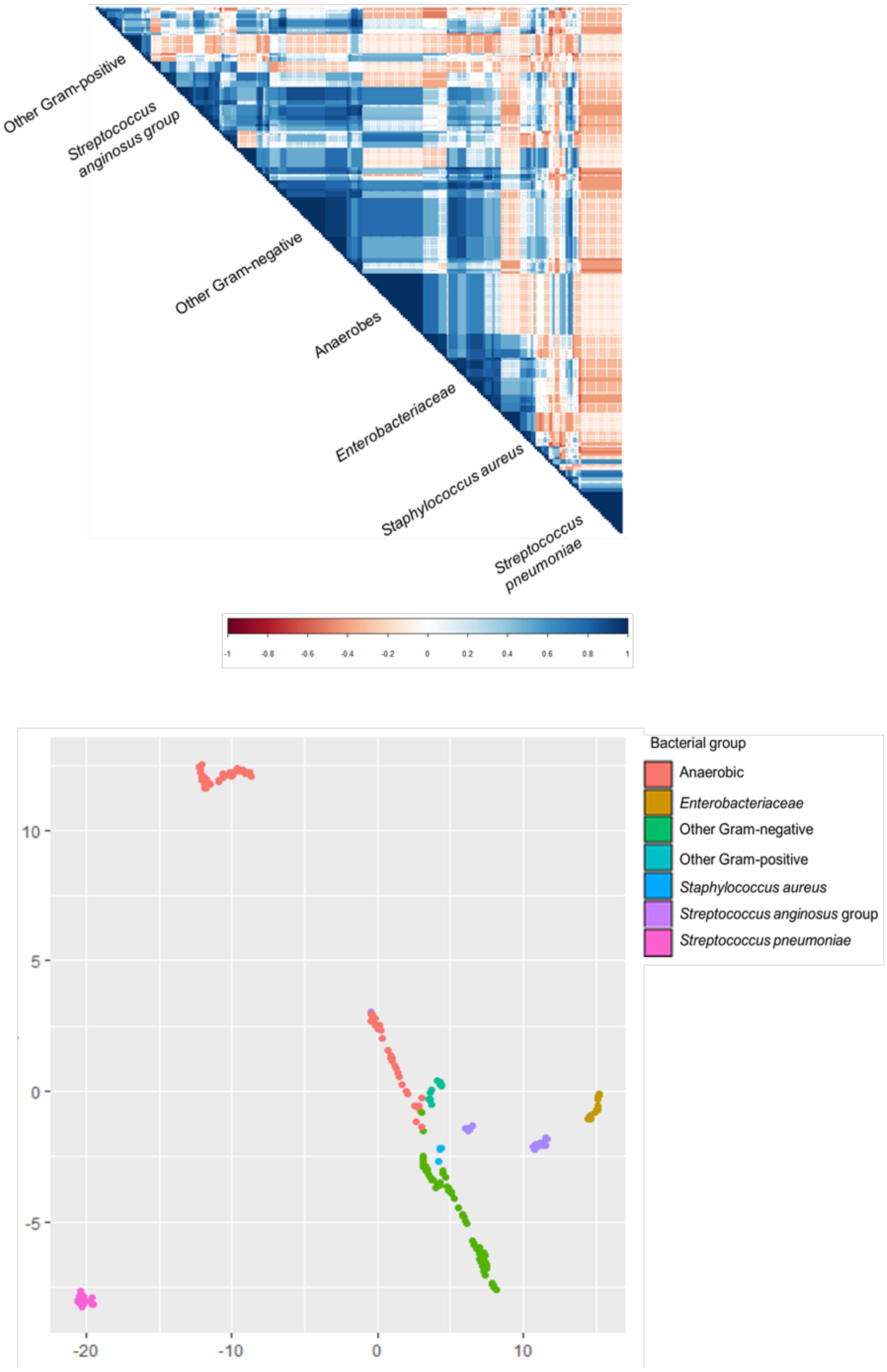

Supplementary Figure 2. Diverse bacterial patterns were detected in pleural infection samples. (A) Correlation plot of the association between the samples using the Spearman method. Dark red denotes negative and dark blue positive correlation. A positive correlation was detected among anaerobes and other Gram-negative

bacteria. **(B)** Uniform Manifold Approximation and Projection (UMAP) graph for the PILOT cohort. The samples are coloured by the dominant bacterial group. Samples predominated by Streptococcus pneumoniae showed narrower spread compared with the other groups, implying a closer relationship. UMAP clustered samples dominated by anaerobic bacteria into two clusters, the first distant and the latter close to samples dominated by other Gram-negative bacteria. The first cluster denotes the samples predominately consisted of anaerobes and the second cluster samples with a mixed population of anaerobes and other Gram-negative bacteria.

Supplementary Figure 3

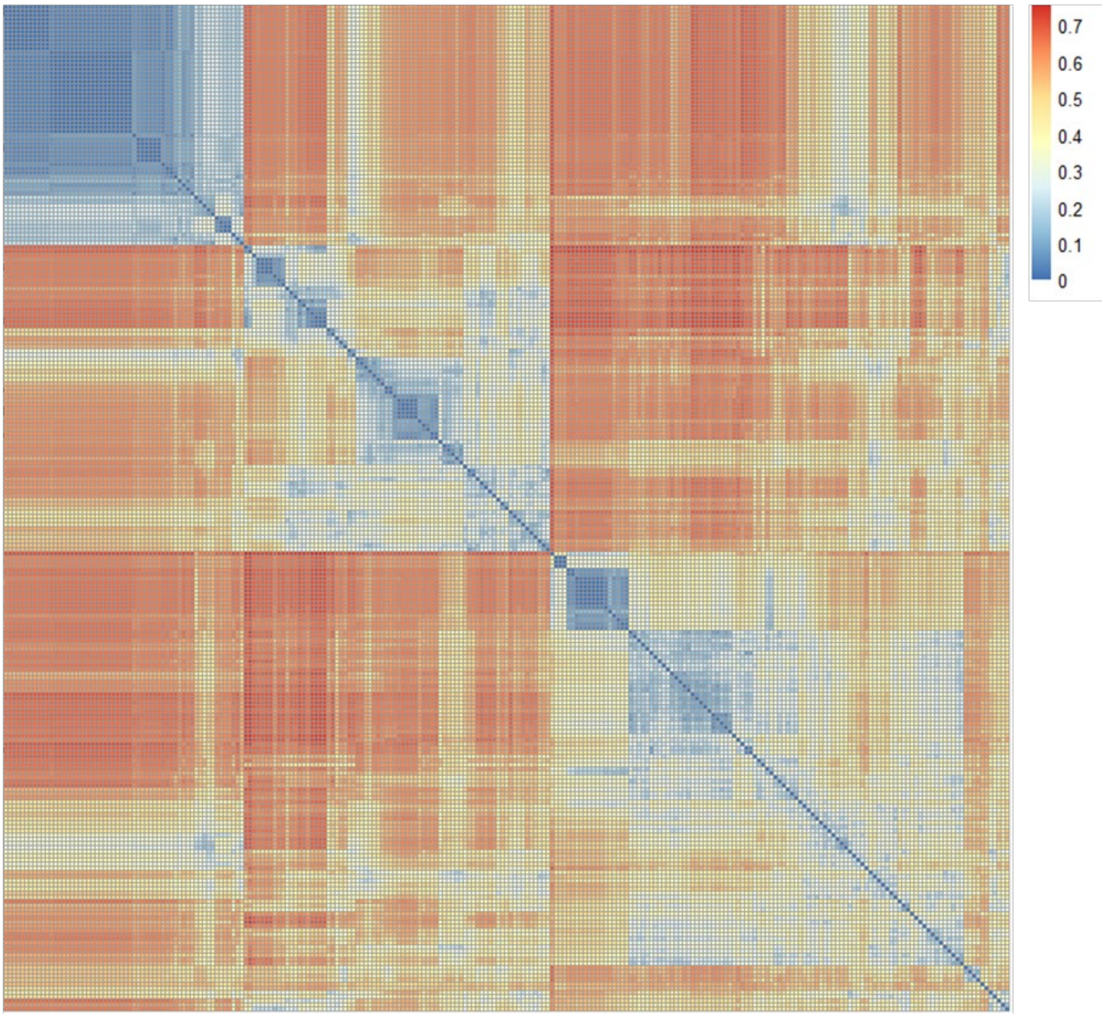

Supplementary Figure 3. Distance analysis comparing the microbiology between the samples.
Correlation plot presenting the distance between the samples (beta diversity). Different microbiological communities were detected by the weighted UniFrac distance metric. Dark blue shows high similarity whereas, far red high diversity.

Supplementary Figure 4

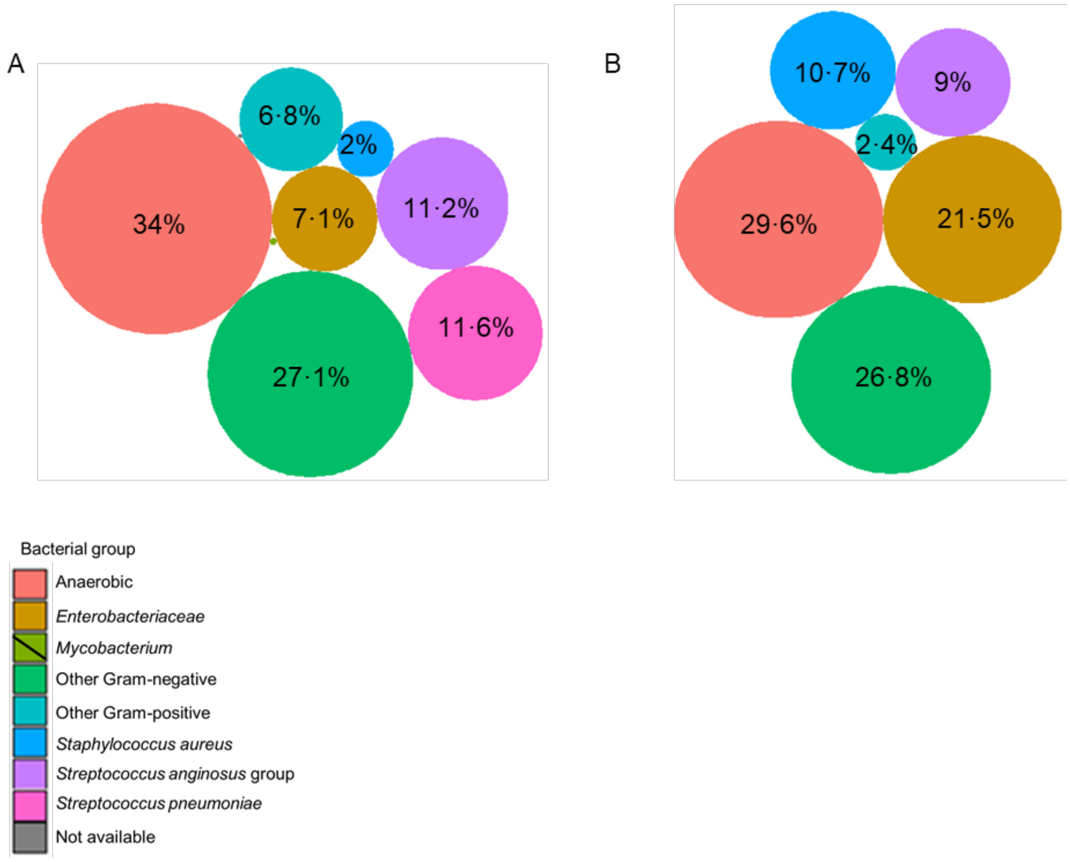

Supplementary Figure 4. Distinct bacterial patterns were detected amond community and hospital acquired pleural infection. The identified bacteria were classified into nine groups (Table 1). **(A-B)** Circle packing graphs presenting the abundance (percentage of bacterial reads) of each bacterial class for **(A)** community and **(B)** hospital acquired pleural infection (Supplementary Tables 4 and 5).

Supplementary Figure 5

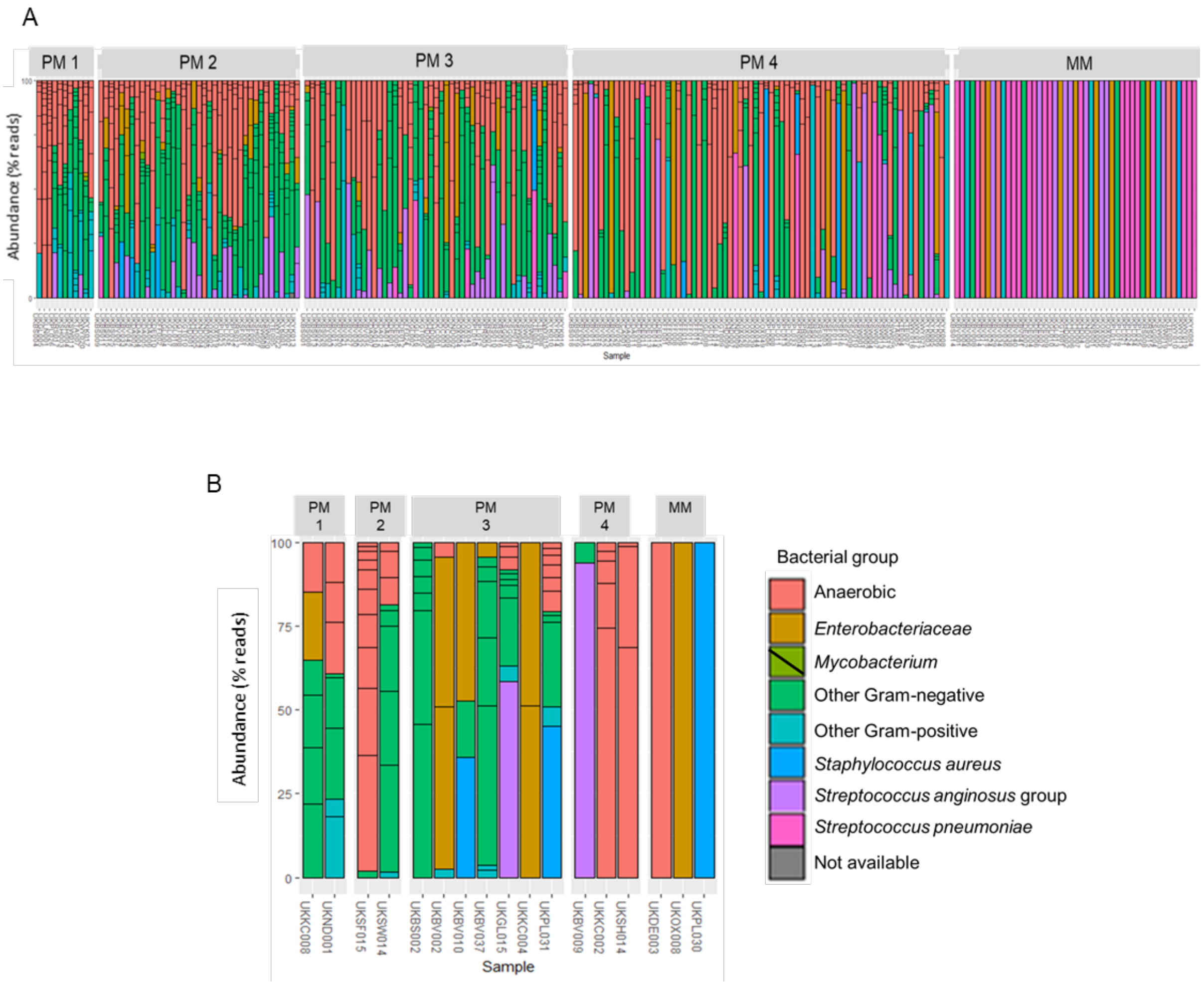

Supplementary Figure 5. The bacteriology of community and hospital acquired pleural infection is diverse. (A-B) Bar plots representing the abundance (% of reads per sample) of the bacterial classes which were identified in each sample for **(A)** community- **(B)** hospital-acquired pleural infection. The samples have been grouped based on the maximum relative abundance of their pathogens. The colours of the bars represent the bacterial classes.

Supplementary Figure 6

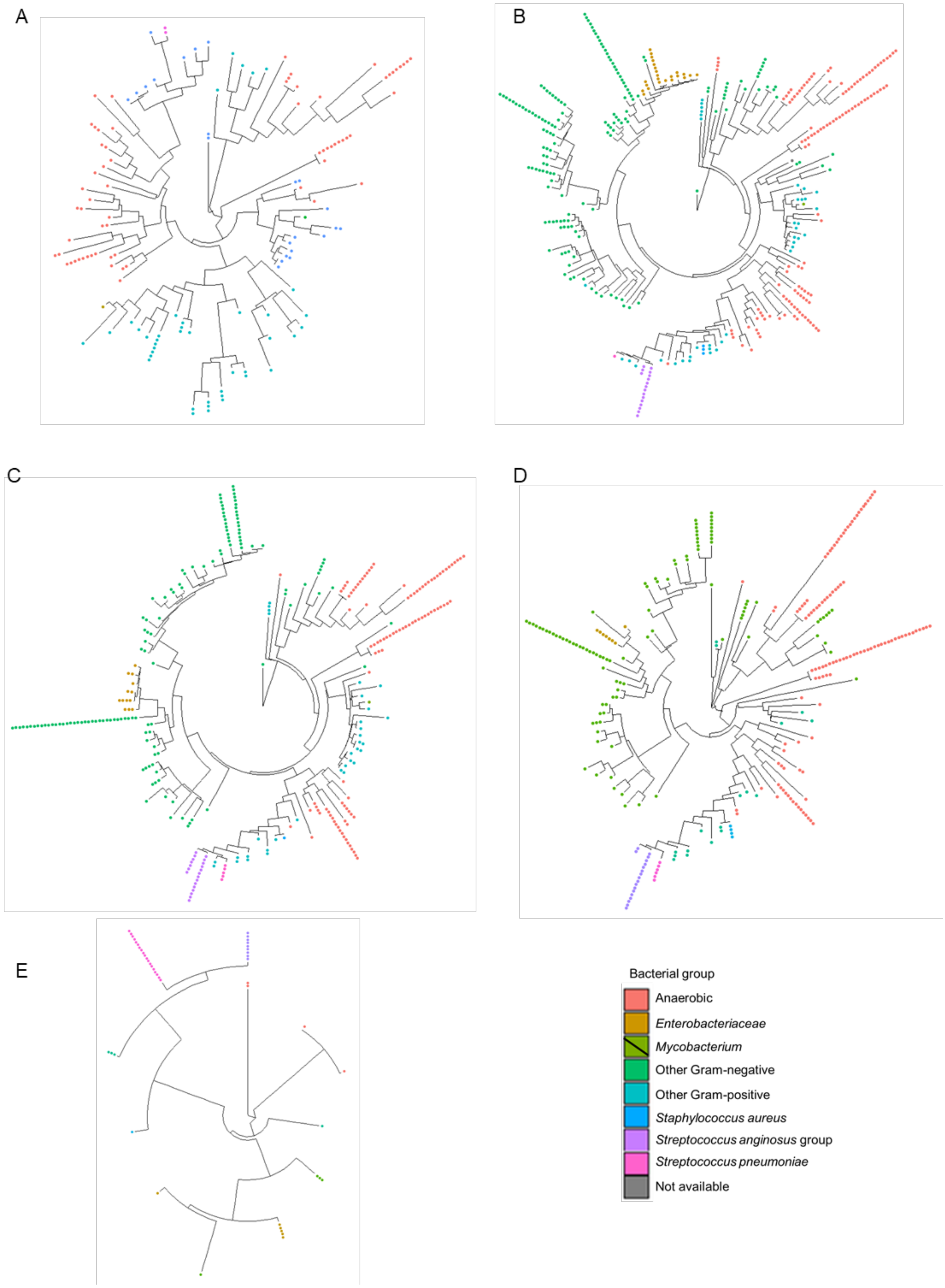

Supplementary Figure 6. Community acquired polymicrobial pleural infection exhibit higher species richness than monomicrobial infections.
(A-E) Phylogeny trees for **(A)** polymicrobial group 1 (0, 25%), **(B)** group 2 [25, 40%), **(C)** group 3 [40, 60%) **(D)** group 4 [60, 100%) and **(E)** monomicrobial group [100%] community acquired pleural infection. Polymicrobial groups 1-4 exhibited a bacterial population of greater species richness (Supplementary Table 3) compared to the monomicrobial group.

Supplementary Figure 7

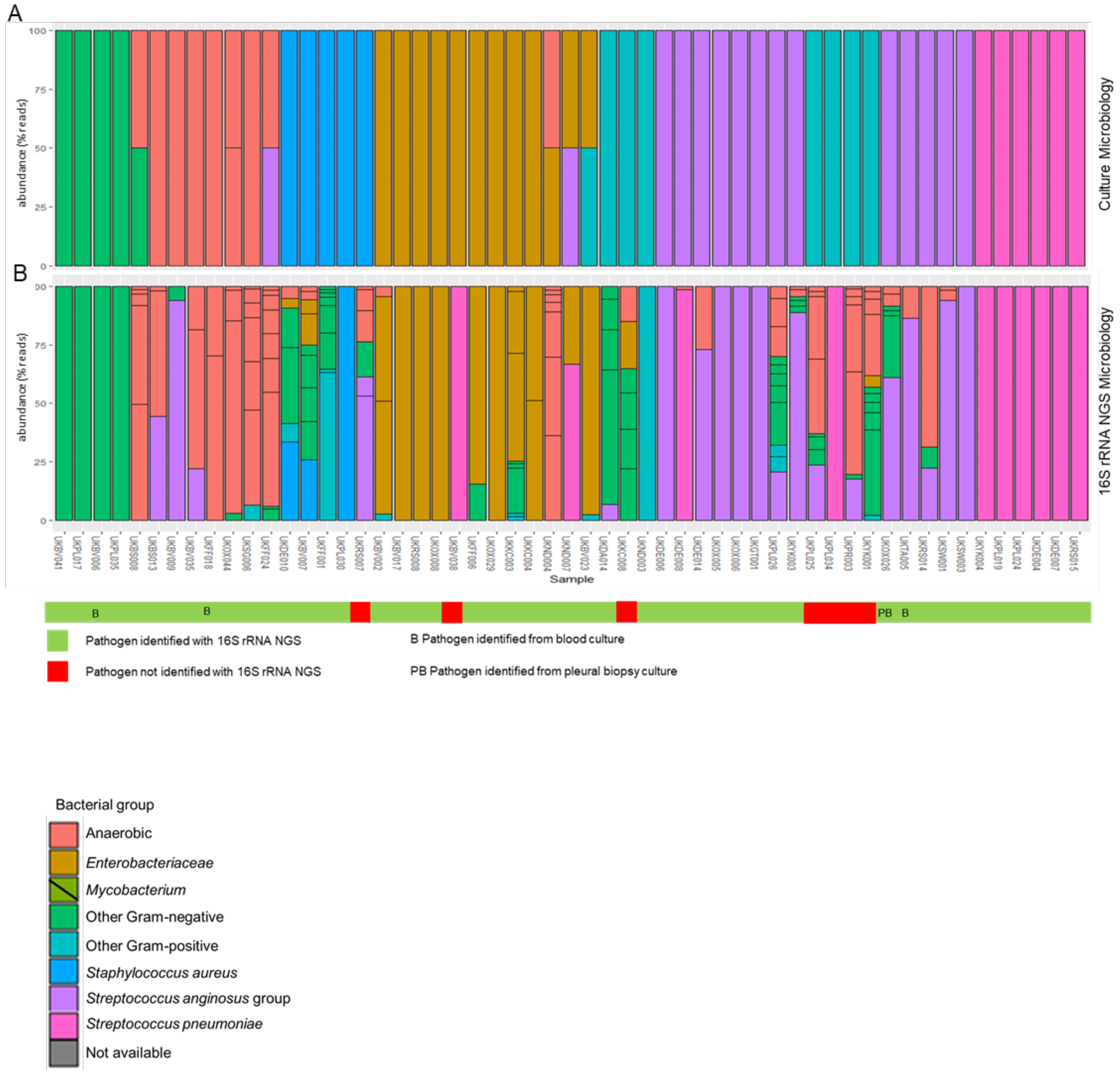

Supplementary Figure 7. Comparison between culture and 16S rRNA NGS microbiology.

Bar plots showing the bacterial groups which were detected in the culture positive samples by **(A)** culture and **(B)** 16S rRNA NGS. In total 55 (22%) samples had positive culture microbiology. In average culture detected 1·1 (SD 0·3) pathogens while 16S rRNA NGS had a higher yield of 3·2 (SD 2·8) pathogens per sample. 16S rRNA NGS identified the pathogen detected by culture for 48 (87%) samples. For seven samples the findings between the two techniques did not agree. However, there were five samples that 16S rRNA NGS detected a pathogen with the same taxonomy up to the genus level as culture.

Supplementary Figure 8

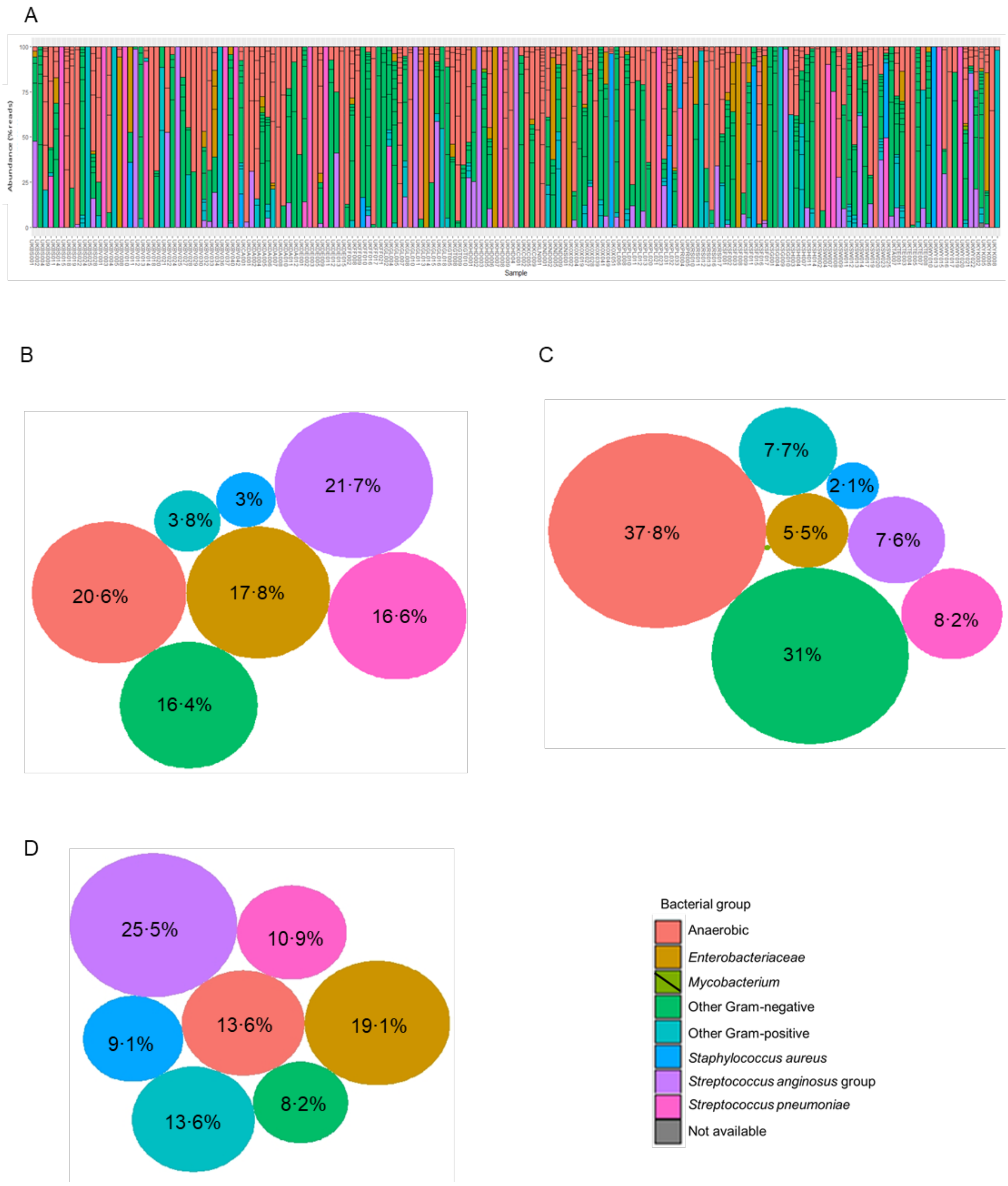

Supplementary Figure 8. Comparison between positive and negative culture microbiology.
(A) Bar plot showing the abundance (% of reads per sample) of the bacterial groups which were detected in each of the culture negative samples by 16S rRNA NGS. In total 188 (78%) samples had negative culture microbiology. **(B-C)** Circle packing graphs presenting the abundance (percent of bacterial reads) of each bacterial class

for **(B)** positive and **(C)** negative culture samples by 16S rRNA. **(D)** Circle packing graph presenting the abundance based on clinical microbiology results. Bacteria of the *Streptococcus anginosus* group were the most frequently detected pathogens by culture.

Supplementary Figure 9

A

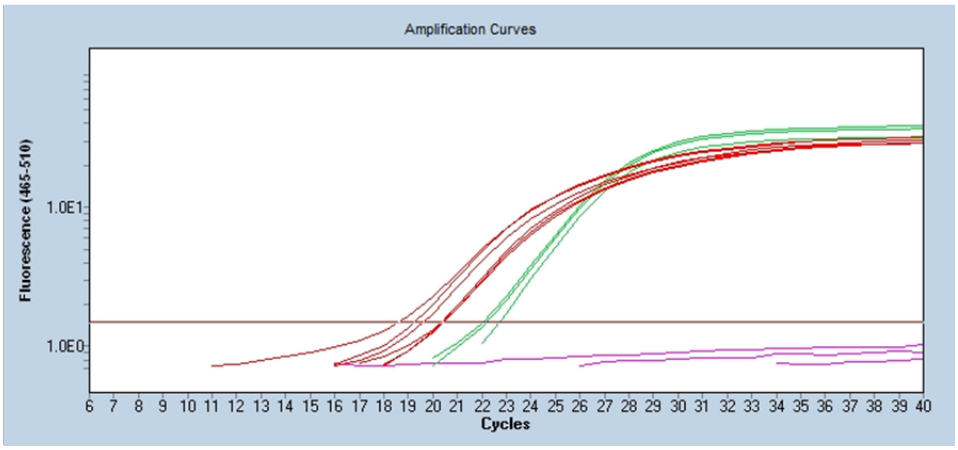

B

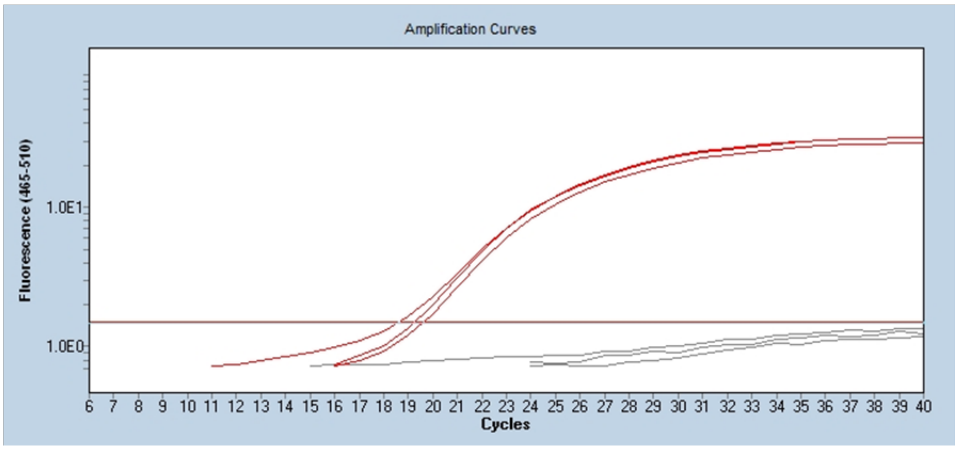

Supplementary Figure 9. Verification of 16S rRNA NGS data by qPCR assays. For two of the samples 16S rRNA NGS did not detect the pathogens which were identified by culture. 16S rRNA NGS classified the first sample as monomicrobial with *Streptococcus pneumoniae* while culture detected *Serratia marcescens*. 16S rRNA NGS categorised the second sample as polymicrobial without identifying *Micrococcus luteus* which was the culture finding. We performed qPCR assays to cross examine the culture and 16S rRNA NGS results. **(A)** Amplification curves for 16S rRNA (red, universal), *lytA* (green, *Streptococcus pneumoniae* specific) and *luxS* (pink, *Serratia marcescens* specific) bacterial genes. qPCR detected *Streptococcus pneumonia* but not *Serratia marcescens*. **(B)** Amplification curves for *16S* rRNA (red, universal) and *Rpf* (grey *Micrococcus luteus*) bacterial genes. qPCR did not detect the presence of *Micrococcus luteus*.

Figure 10

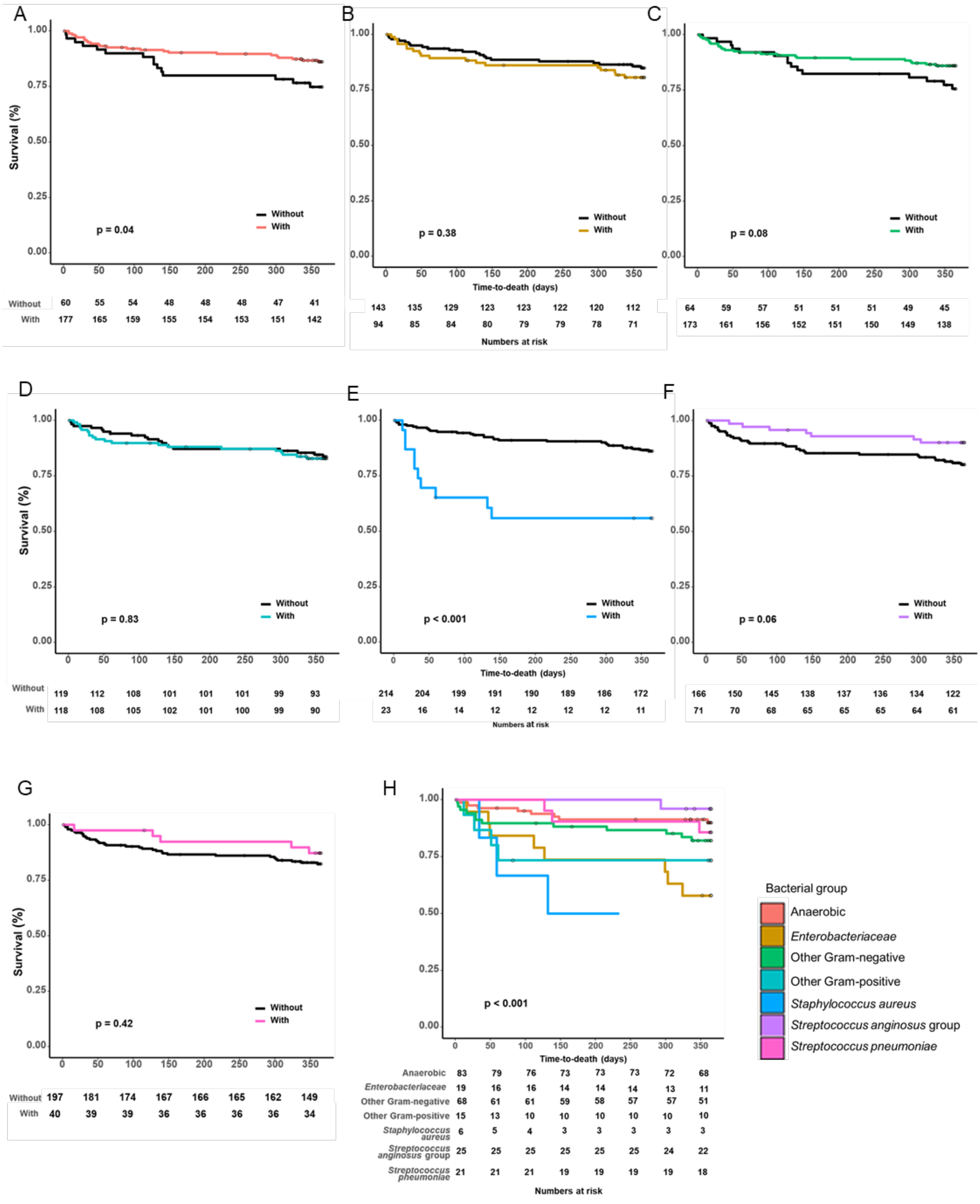

Supplementary Figure 10. The association between one-year survival and bacterial patterns. (A-H) Kaplan-Meier survival curves presenting one-year

mortality. **(A-G)** The samples were separated based on the presence or absence of **(A)** anaerobes, **(B)** Enterobacteriaceae, **(C)** other Gram-negative, **(D)** other Gram-positive, **(E)** Staphylococcus aureus, **(F)** Streptococcus anginosus group and **(G)** Streptococcus pneumoniae. The black line in each graph represents all other samples. The one-year mortality outcomes were analysed using multivariate Cox regression analyses, adjusting for the factors of the RAPID score. Microbiology was an independent risk factor of mortality. **(H)** The samples were separated based on the dominant bacterial group in each sample. Samples with dominance in Enterobacteriaceae and Staphylococcus aureus exhibited poorer survival and increased risk of one-year mortality. In the multivariate Cox regression analyses patients with missing data for the RAPID score were excluded from the analyses, resulting in the exclusion of 33 patients.

Supplementary Figure 11

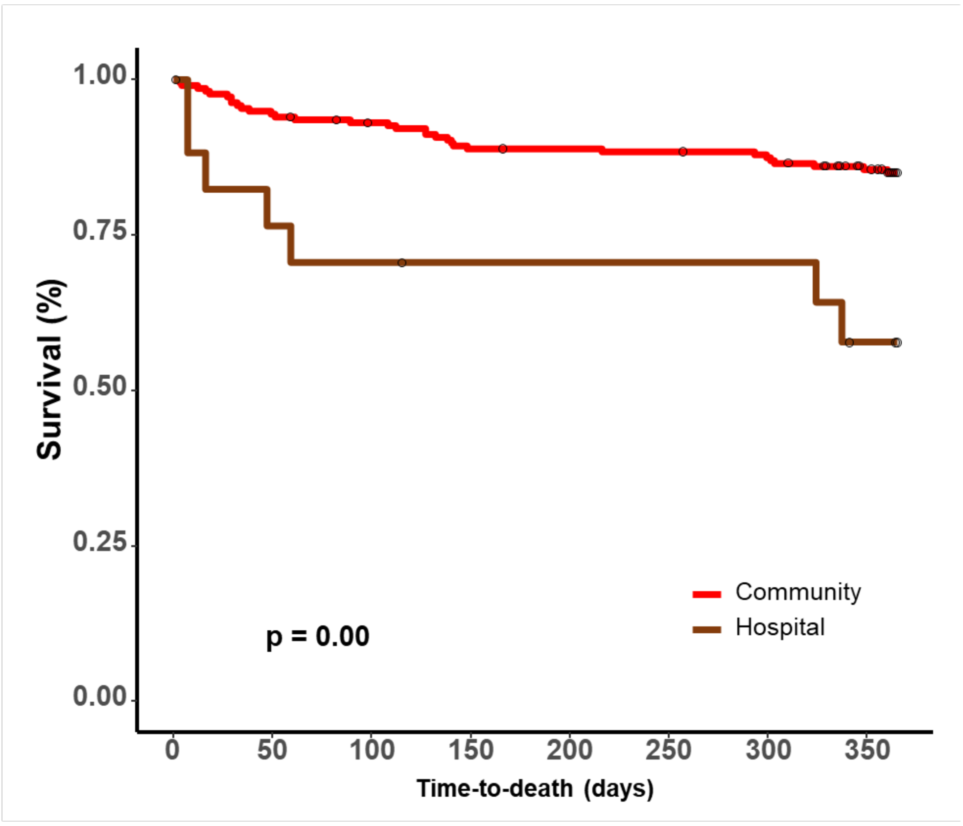

Community	219	206	200	191	190	189	186	174
Hospital	17	13	12	11	11	11	11	8

Numbers at risk

Supplementary Figure 11. Hospital acquired pleural infection exhibited poorer survival compared to community acquired pleural infection.
Kaplan-Meier survival curves presenting one-year mortality for community and hospital acquired pleural infection (Multivariate Cox regression P value). The increased pathogenic and antibiotic resistance seen with nosocomial pathogens is associated with shorter survival. In the multivariate logistic regression analyses patients with missing data for the RAPID score were excluded from the analyses, resulting in the exclusion of 33 patients.

Supplementary Figure 12

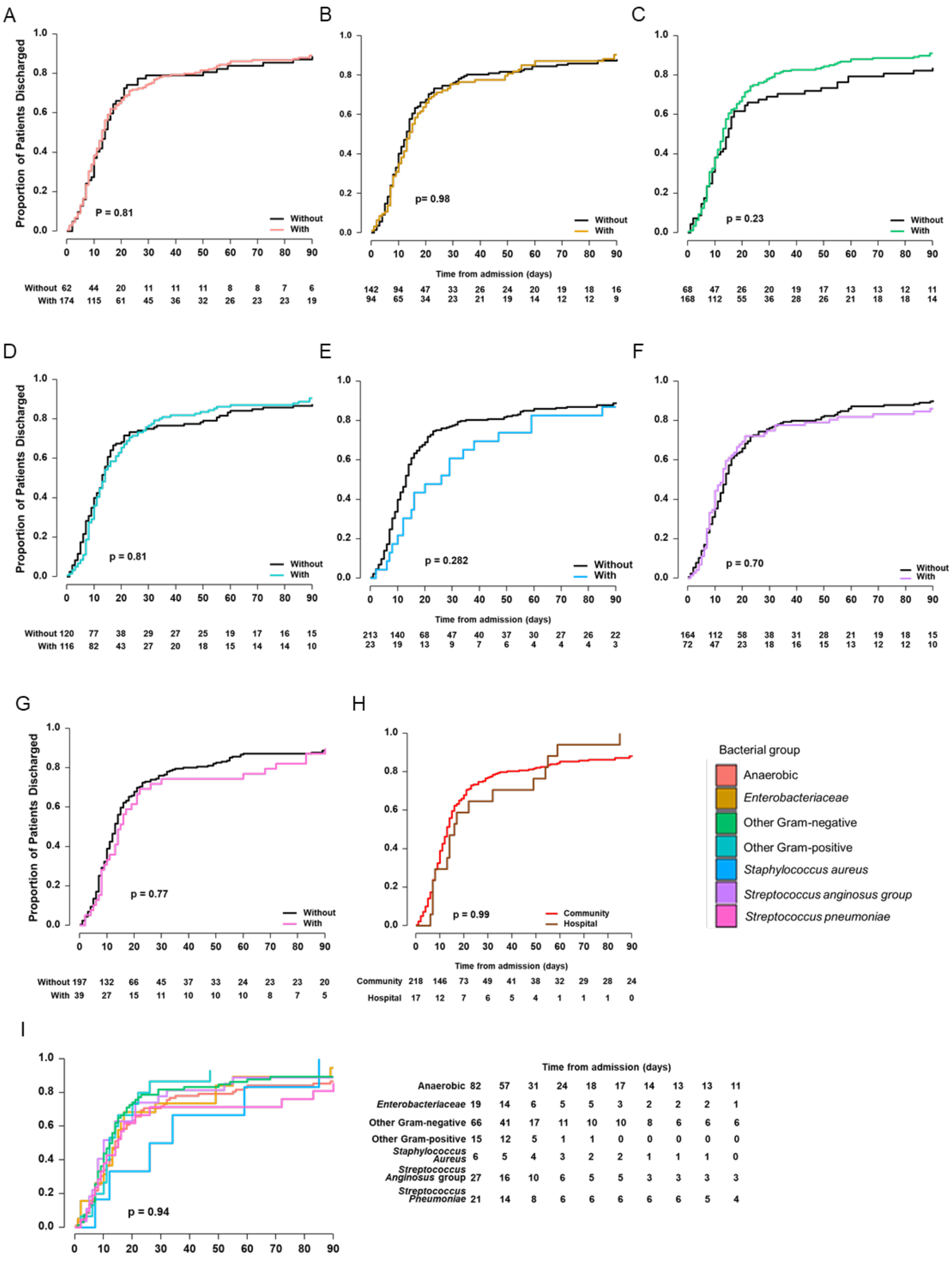

Supplementary Figure 12. No association was detected between the duration of hospitalisation and bacterial patterns.
(A-I) Cumulative incidence plots for length of stay. The plots account for the competing risk of dying. **(A-G)** The samples have been separated based on the presence/absence of **(A)** anaerobes, **(B)** *Enterobacteriaceae*, **(C)** Other Gram-negative, **(D)** other Gram-positive, **(E)** *Staphylococcus aureus*, **(F)** *Streptococcus anginosus* group and **(G)** *Streptococcus pneumoniae*. **(H-I)** The samples have been categorised based on **(H)** infection acquisition place and **(I)** the dominant bacteria group. In the multivariate Cox regression analyses patients with missing data for the

RAPID score were excluded from the analyses, resulting in the exclusion of 33 patients.

References

1. Psallidas, I., *et al.* A Pilot Feasibility Study in Establishing the Role of Ultrasound-Guided Pleural Biopsies in Pleural Infection (The AUDIO Study). *Chest* **154**, 766-772 (2018).
2. Klindworth, A., *et al.* Evaluation of general 16S ribosomal RNA gene PCR primers for classical and next-generation sequencing-based diversity studies. *Nucleic Acids Res* **41**, e1 (2013).
3. Simon, A. FastQC: a quality control tool for high throughput sequence data. (2010).
4. Callahan, B.J., *et al.* DADA2: High-resolution sample inference from Illumina amplicon data. *Nat Methods* **13**, 581-583 (2016).
